# Global estimation of anti-malarial drug effectiveness for the treatment of uncomplicated *Plasmodium falciparum* malaria 1991–2019

**DOI:** 10.1186/s12936-020-03446-8

**Published:** 2020-10-20

**Authors:** Giulia Rathmes, Susan F. Rumisha, Tim C. D. Lucas, Katherine A. Twohig, Andre Python, Michele Nguyen, Anita K. Nandi, Suzanne H. Keddie, Emma L. Collins, Jennifer A. Rozier, Harry S. Gibson, Elisabeth G. Chestnutt, Katherine E. Battle, Georgina S. Humphreys, Punam Amratia, Rohan Arambepola, Amelia Bertozzi-Villa, Penelope Hancock, Justin J. Millar, Tasmin L. Symons, Samir Bhatt, Ewan Cameron, Philippe J. Guerin, Peter W. Gething, Daniel J. Weiss

**Affiliations:** 1 Malaria Atlas Project, Big Data Institute, Nuffield Department of Medicine, University of Oxford, Oxford, UK; 2 WorldWide Anti-Malarial Resistance Network (WWARN), Oxford, UK; 3 Infectious Diseases Data Observatory (IDDO), Oxford, UK; 4 Centre for Tropical Medicine and Global Health, Nuffield Department of Clinical Medicine, University of Oxford, Oxford, UK; 5 Institute for Disease Modeling, Bellevue, WA USA; 6 Imperial College London, London, UK; 7 Telethon Kids Institute, Perth, Australia; 8 Curtin University, Perth, Australia; 9 Center for Data Science, Zhejiang University, Hangzhou, 310058 China

**Keywords:** Falciparum malaria, Anti-malarial drug effectiveness, Drug quality, Global

## Abstract

**Background:**

Anti-malarial drugs play a critical role in reducing malaria morbidity and mortality, but their role is mediated by their effectiveness. Effectiveness is defined as the probability that an anti-malarial drug will successfully treat an individual infected with malaria parasites under routine health care delivery system. Anti-malarial drug effectiveness (AmE) is influenced by drug resistance, drug quality, health system quality, and patient adherence to drug use; its influence on malaria burden varies through space and time.

**Methods:**

This study uses data from 232 efficacy trials comprised of 86,776 infected individuals to estimate the artemisinin-based and non-artemisinin-based AmE for treating falciparum malaria between 1991 and 2019. Bayesian spatiotemporal models were fitted and used to predict effectiveness at the pixel-level (5 km × 5 km). The median and interquartile ranges (IQR) of AmE are presented for all malaria-endemic countries.

**Results:**

The global effectiveness of artemisinin-based drugs was 67.4% (IQR: 33.3–75.8), 70.1% (43.6–76.0) and 71.8% (46.9–76.4) for the 1991–2000, 2006–2010, and 2016–2019 periods, respectively. Countries in central Africa, a few in South America, and in the Asian region faced the challenge of lower effectiveness of artemisinin-based anti-malarials. However, improvements were seen after 2016, leaving only a few hotspots in Southeast Asia where resistance to artemisinin and partner drugs is currently problematic and in the central Africa where socio-demographic challenges limit effectiveness. The use of artemisinin-based combination therapy (ACT) with a competent partner drug and having multiple ACT as first-line treatment choice sustained high levels of effectiveness. High levels of access to healthcare, human resource capacity, education, and proximity to cities were associated with increased effectiveness. Effectiveness of non-artemisinin-based drugs was much lower than that of artemisinin-based with no improvement over time: 52.3% (17.9–74.9) for 1991–2000 and 55.5% (27.1–73.4) for 2011–2015. Overall, AmE for artemisinin-based and non-artemisinin-based drugs were, respectively, 29.6 and 36% below clinical efficacy as measured in anti-malarial drug trials.

**Conclusions:**

This study provides evidence that health system performance, drug quality and patient adherence influence the effectiveness of anti-malarials used in treating uncomplicated falciparum malaria. These results provide guidance to countries’ treatment practises and are critical inputs for malaria prevalence and incidence models used to estimate national level malaria burden.

## Background

The Global Burden of Disease (GBD) study estimated that 619,800 (95% uncertainty intervals: 440,100–839,500) malaria deaths occurred worldwide in 2017 [1], with over 80% of deaths occurring in sub-Saharan Africa [2]. Malaria mortality has decreased substantially over the last two decades through increased investment in the availability of effective treatments, such as artemisinin-based combination therapy (ACT) and preventive measures such as long-lasting insecticidal nets [3–5]. Anti-malarial treatments are key to curbing malaria burden and mortality as they reduce the individual’s risk of severe disease and death in incident cases [6, 7] while also decreasing the infectious reservoir of individuals from where mosquitoes can acquire a blood meal. Reaching global malaria reduction targets requires a detailed understanding of current treatment coverage levels as well as factors limiting their effectiveness. Coverage of anti-malarial effectiveness in the population necessitates that: (i) all patients with confirmed malaria infection access an anti-malarial treatment; (ii) the provided drug is of high efficacy and good quality; and, (iii) all patients receive an optimal dosing and adhere to the treatment regimen. This covers all relevant providers involved in managing malaria patients, including private sector, both formal and informal. Whilst efficacy, which drives most of the drug policy decisions, is primarily dictated by the evolution of drug-resistant parasite phenotypes, anti-malarial drug effectiveness (AmE) is a composite measure that encompasses clinical efficacy (the performance of the medicine under controlled conditions) and other real-world clinical practice limitations. AmE is influenced by: (i) patient-specific responses to the treatment, including absorption, genetics, co-morbidities, special conditions such as pregnancy, very young age, or drug-drug interactions; (ii) patient adherence to the drug’s use instructions; (iii) healthcare providers’ skill, knowledge and prescription practises; (iv) health system performance; (v) access to the healthcare system; (vi) healthcare expenditure; and, (vii) other socio-demographic characteristics that limit the appropriate use of anti-malarial drugs.

The World Health Organization (WHO) recommends the use of ACT for treatment of uncomplicated falciparum malaria [8], as this species causes the most severe forms of malaria and subsequently death. Currently, ACT is used by most malaria-endemic countries and territories [9, 10] as first-line treatment for falciparum malaria (Additional file 1: Section 1), and its use has been widespread. For example, 82% of febrile children treated with an anti-malarial received an ACT medicine in public health facilities within sub-Saharan Africa between 2015 and 2018 [11]. High efficacy of ACT for *Plasmodium falciparum* infection has been reported in sub-Saharan Africa, with no artemisinin resistance confirmed in this region [12]. Historically, anti-malarial resistance has been a major obstacle in the fight against malaria [3, 12–15]. Chloroquine (CQ) resistance was first observed in Southeast Asia and in South America in the late 1950s, and later spread to Africa. It was replaced by sulfadoxine-pyrimethamine (SP), to which resistance quickly emerged and spread from Southeast Asia to most endemic areas, and the drug became ineffective [16]. Evidence supporting the high efficacy and safety of artemisinin derivatives when paired with a partner drug became available in the late 1990s [17–19]. However, the emerging artemisinin resistance reported since the mid-2000s poses a significant threat to the recent gains in malaria control. Resistance to artemisinin was first identified in the Greater Mekong Sub-region (GMS), and there are concerns that it has spread to densely populated countries like India and recently to parts of sub-Saharan Africa [3, 14, 20–30]. The historical use of artemisinin as a monotherapy [12, 14, 15], or the use of partner drugs with similar modes of action and cross-resistance [31, 32], are among the primary factors fuelling the development of resistance. Other potential factors include the use of sub-standard and falsified medicines, high prevalence of self-treatment, poor adherence to drug use protocols, weak healthcare systems, and unmonitored treated cases [33–37]. All these underscore the need for a full understanding of the spatiotemporal pattern of ACT effectiveness in all *P. falciparum* endemic countries. Likewise, the effectiveness of non-artemisinin anti-malarials remains important as they are still first-line treatments for falciparum malaria in some countries [11] and widely used in many areas despite adaptation of WHO recommendations [12]. Together the ACT and non-ACT results from this research will provide crucial information for assessing the impact of effectiveness on falciparum malaria burden, monitoring emerging resistance (including multi-drug resistance), and better characterizing anti-malarial drug distribution, quality, access, and use [17, 37–40]. When it comes to saving lives, effectiveness of drugs used for treatment is a key control intervention, and the need to characterize changing patterns of AmE is evident [41]. A global assessment of anti-malarial drug efficacy was published in 2010; existence of recently conducted and published anti-malarial efficacy trials [42] emphasizes the rationale for deriving updated global AmE estimates that will inform researchers, stakeholders and countries’ malaria control programmes [14, 20, 43–46].

To precisely monitor and compare AmE over space and time, and at a global scale, standardized data and methodologies are required. Clinical efficacy data are a key input for such analyses, and several databases cataloguing this metric have been established. As the databases were created for differing research purposes, they have inconsistent structures and varying foci, including specific drugs, regions or time periods [44, 47–56] (Additional file 1: Section 1). Most of these databases do not provide patient-specific data to support further statistical analysis, and contain location-based drug efficacy estimates derived using different methodologies, in both design and analysis [48]. The WorldWide Anti-malarial Resistance Network (WWARN) responded to the challenge of comparing varying anti-malarial drug efficacy estimates by: (i) acquiring individual-patient-level data from efficacy trials conducted and published since 1960 [57]; (ii) re-analysing the patient-level data using a consistent methodology (modified Intention-To-Treat analysis); and, (iii) using standardized indicator definitions to produce comparable drug efficacy estimates [58, 59]. The result of WWARN’s work is the most comprehensive, standardized and accessible anti-malarial drug efficacy database yet created. Critically, WWARN dataset provides comparable results within and between countries, and over time thus supporting a spatiotemporal analysis.

Clinical drug efficacy trials suffer from various complications, including non-compliance, protocol withdrawals and deviations (e.g., co-morbidity, exposure to new infections and health worker mistakes) that may result in participants being dropped during the analysis phase. The Intention-To-Treat analytical approach is advantageous because it includes all study participants according to the initial randomization, regardless of deviations from the protocol, such as participant withdrawals from the study or re-infection [59–61]. This method gives more conservative and unbiased efficacy estimates that are closer to what would happen in clinical practice and are proxy for effectiveness [62–64] (study endpoints by WHO and WWARN—Additional file 1: Section 1). There are deficiencies in existing routine health information systems for adequately monitoring responses to malaria treatment, and few studies have assessed effectiveness of anti-malarials globally. As such, analysing WWARN estimates within geospatial models that (i) include health system, socio-demographic and environmental factors, while (ii) adjusting for adherence and quality of drugs, provides a reasonable basis for deriving measures of AmE [65]. Furthermore, the covariates introduce information linked to effectiveness rather than efficacy, thereby allowing the model to amplify or reduce the gap between efficacy and effectiveness based on local conditions.

This analysis generates fine-scale, global temporally dynamic maps of AmE for uncomplicated falciparum malaria. This metric defines the treatment success rate of an anti-malarial drug when administered to *P. falciparum-*infected individuals under typical use conditions (e.g., drugs obtained from facilities or pharmacies). This considers all individuals with parasitological- or clinically confirmed malaria infections that subsequently received an anti-malarial drug for treatment, regardless of whether they had a malaria-attributable fever, a fever attributable to a co-infection, or no fever. Effectiveness is estimated for both artemisinin-based and non-artemisinin-based treatment in all malaria-endemic countries from 1991 to 2019. The AmE models include covariates for health system factors, climate and environmental variables, socio-demographic, malaria transmission risks, and population. The estimates are adjusted for quality of anti-malarial drugs and adherence to dosage regimens [33, 66]. The rationale for modelling AmE rather than clinical efficacy is threefold: (i) effectiveness is a more relevant metric for assessing anti-malarial impacts when administered within a real-world clinical setting (i.e., after it becomes a front-line treatment for malaria within a country); (ii) modelling effectiveness allows use of spatially varying covariates that can feasibly be related to effectiveness but are unlikely to influence efficacy; and, (iii) effectiveness is an essential input for calculating malaria mortality in the GBD study. The GBD study includes annual, national-level estimates of morbidity and mortality attributed to malaria, with accompanying high-spatial-resolution (5 × 5 km) maps produced by the Malaria Atlas Project (MAP) [67, 68]. Prior to use in modelling malaria burden, the results of this research (AmE) are combined with proportional anti-malarial use and treatment-seeking rates to generate estimates of effective treatment with anti-malarials, which are then used for modelling *P. falciparum* prevalence and estimating the proportion of cases that are not successfully treated.

## Methods

### Data

The response data used in this research consisted of 232 anti-malarial drug efficacy studies conducted between 1991 and 2016, comprising 756 treatment arms, and information on 86,776 individuals. Among the studies used for analysis, 203 were extracted from the WWARN database, and comparable metrics were extracted from additional studies (n = 29) obtained through a review of articles assembled in the WWARN clinical trials publication library [61]. For consistency, these additional studies were selected using the same criteria applied when generating the WWARN dataset (i.e., additional studies focused on treatment of falciparum malaria, did not include pregnant women, and used a modified Intention-To-Treat approach). The WWARN database consists of publicly available aggregated results harmonized and summarized from anti-malarial drug efficacy studies, including both artemisinin-based (including artesunate monotherapy) and non-artemisinin-based therapy (Additional file 1: Section 1). Information of the trials included in the analysis is presented in Table 1. Additional file 1: Section 2 illustrates distribution and number of studies by treatment type, country and year.

**Table 1:** Characteristics of anti-malarial drug efficacy trials

	Africa	Asia	South America	Total^a^
Number of ACT-studies (%)	133 (69.4)	65 (28.0)	6 (2.6)	204
Number of non-ACT-studies (%)	62 (100)	0	0	62
Number of subjects (%)	64,553 (74.4)	21,168 (24.4)	1,055 (1.2)	86,776
Study year, range	1993–2015	1991–2016	2004–2016	1993–2016
Age at enrolment (no of studies)				
Under 5 years of age	67	2	0	69
All ages	128	63	6	197
Treatment studied (no of treatment arms in efficacy study)				
ACT (including monotherapy)				
Artesunate	21	24	1	46
Artemether–lumefantrine (AL)	169	32	2	203
Artesunate–amodiaquine (ASAQ)	94	6	1	101
Artesunate–mefloquine (ASMQ)	15	62	3	80
Artesunate–sulfadoxine–pyrimethamine (ASSP)	41	47	0	88
Dihydroartemisinin piperaquine (DHAP)	50	53	1	104
Non-ACT				
Chloroquine (CQ)	35	0	0	35
Sulfadoxine–pyrimethamine (SP)	69	0	0	69
Others	30	0	0	30
Efficacy %, median				
ACT (including monotherapy)	97.9	100	100	99.3
Artesunate	86.4	100	100	95.5
Artemether–lumefantrine	97.8	97.4	100	98.4
Artesunate–amodiaquine	97.8	94.9	97.1	96.6
Artesunate–mefloquine	99.3	99.2	100	99.5
Artesunate–sulfadoxine–pyrimethamine	97.7	100		98.9
Dihydroartemisinin piperaquine	98.4	100	99.2	99.2
Non-ACT	83.1			83.1
Chloroquine	61.1			61.1
Sulfadoxine–pyrimethamine	91.3			91.3
Others	82.3			82.3

^a^Some studies assessed both drugs, hence counted twice

### Covariates

Covariates used for modelling were identified from several procedures. First, publications describing factors influencing treatment failure, effectiveness of anti-malarials, quality of anti-malarials, anti-malarial usage, and adherence, were identified with a literature review that followed an established methodology [69]. Second, a comprehensive search of the WWARN clinical trials publication library, which includes all available and published anti-malarial efficacy studies, was performed. Papers were considered if they studied *P. falciparum* only, did not include pregnant women, used a modified Intention-To-Treat statistical analysis approach, and had a follow-up time of at least 28 days. A total of 733 out of 1178 publications that reported efficacy matched these criteria, and all these were reviewed to extract information on covariates related to anti-malarial efficacy. Third, using similar criteria, an additional 47 papers focusing on malaria risk mapping were identified from publication databases and included for review. This literature review identified factors related to malaria infection, transmission and risk that are relevant when assessing the performance of anti-malarial drugs in real-world settings. Finally, grey literature, including WHO Malaria reports, was examined. The databases searched were PubMed, Embase, the Web of Science library, and Google Scholar. All covariates mentioned as having an influence on anti-malarial drug efficacy, malaria treatment effectiveness or malaria infection in the compiled literature were identified. Additional file 1: Section 3—Table S3.1 presents some of literature reviewed, identified factors and their importance to this analysis.

The Institute for Health Metrics and Evaluation (IHME) [70] and MAP [71] provided the covariates used in this analysis [69, 72]. IHME collates and produces country- and annual-level variables, including health system access and metrics characterizing socio-economic status. MAP compiles, maintains and generates gridded global covariates characterizing environmental and climatic conditions, which are primarily derived from temporally dynamic and high-resolution satellite images and include temperature, rainfall, vegetation indices, population, night-time lights, and accessibility to cities [69, 72–75]. Data were obtained for 69 factors (Additional file 1: Section 3—Tables S3.2 and S3.3). Six transformations (natural logarithm, reciprocal, squared, cubed, exponential) were applied to all continuous variables to incorporate potential non-linear relationships with the response.

### Variable selection procedures

Following assessment of the collated covariates, 38 out of 53 IHME covariates were dropped as they were unavailable for all countries (e.g., they were only modelled for Africa and not globally). Similarly, 3 out of the 16 MAP covariates were excluded due to incomplete geographical coverage. The selection of the remaining variables was performed separately for artemisinin-based and non-artemisinin effectiveness by fitting generalized linear models with study site-specific random effects. The artemisinin-based and non-artemisinin anti-malarials were modelled separately because (i) the performance of the drug classes had markedly different temporal effectiveness patterns, and (ii) trials on non-artemisinin-based drugs were clustered in Africa, whereas trials on artemisinin-based drugs were performed worldwide. The non-artemisinin-based anti-malarials were combined because too few efficacy studies were available to model them independently. Bivariate analyses relating each response variable with all covariates were performed, and the estimated odds ratios (with associated confidence intervals) and Wald’s p-values were used to assess the significance of the association. This initial process was performed to remove non-significant variables, as the final variable selection was completed later while fitting the geostatistical model. Pearson’s correlation coefficient was used to detect highly correlated covariates. Collinear covariates (r > 0.6) were excluded by ranking them based on their goodness-of-fit, assessed through Akaike Information Criteria (AIC), as well as the residual deviance [76], and removing the less predictive covariates of collinear pairs. Further, multicollinearity was checked by assessing the Variance Inflation Factor (VIF) and performing the Farrar-Glauber test [77]. The selected set of covariates was used in the next steps of model development (Additional file 1: Section 3—Table S3.4).

### Structure of predictive model

The analysis utilized a modelling approach that characterized the spatial process and temporal patterns explicitly to derive predictions and estimate uncertainties [4]. The AmE, for location $k$ and year $j$, ${Y}_{kj}$, was assumed to follow a binomial distribution denoted ${Y}_{kj} \sim Binomial\left({n}_{kj}, {\theta }_{kj}\right)$ where ${n}_{kj}$ was the sample size (the number of patients involved) and ${\theta }_{kj}$ was the probability of treatment success. The relation between the estimates of AmE, covariates, and random effects was modelled via the logit link function:

$$logit\left({\theta }_{kj}\right)= ln\left(\frac{{\theta }_{kj}}{1-{\theta }_{kj}}\right)= {\eta }_{kj}$$

$${\eta }_{kj}={\beta }_{0}+{{\varvec{x}}}_{kj}^{\mathbf{\top }}{\varvec{\beta}}+ {\psi }_{k}+{\xi }_{j},$$

$${\varvec{\beta}}\sim N\left({{\varvec{\mu}}}_{\beta },{{\varvec{\Sigma}}}_{\beta }\right)$$

where ${\eta }_{kj}$ was the linear predictor, ${\beta }_{0}$ the intercept, ${{\varvec{x}}}_{kj}^{\mathbf{\top }}$ was the matrix of covariates and ${\varvec{\beta}}=\left( {\beta }_{1} ,{\beta }_{2}, \dots , {\beta }_{p}\right)$ was the vector of regression coefficients assumed to follow a zero mean Gaussian distribution,${{\varvec{\mu}}}_{\beta }=0$, and a variance${{\varvec{\Sigma}}}_{\beta }$. $\psi ={({\psi }_{1}, \dots {\psi }_{k})}^{T}$ which denotes the spatial random effect which accounted for the spatial dependence. This was assumed to follow a multivariate normal distribution with Matérn covariance function and zero mean (i.e.,$\psi \sim MVN(0,\sum )$). With this specification, the distance at which the spatial correlation is close to 0.1, the spatial range,$r$, and the spatial variance ${\sigma }^{2}$ were estimated. ${\xi }_{j}\sim \mathcal{N}\left(\uprho {\xi }_{j-1},{\tau }^{2}\right),\mathrm{ j}>1$ represent the auto-correlated temporal error terms, while${\xi }_{1}\sim \mathcal{N}\left(0,{\tau }^{2}\right)$; $\tau$ and $\uprho$ are the precision and correlation parameters, respectively. This was a stationary autoregressive process of order one (i.e., AR1), with a Markovian temporal structure, in which the AmE of year $j-1$ influenced the AmE in year $j$. Inclusion of the independent and identically distributed (i.i.d.) site-specific random effects was tested in all model formulations and assessed to see if it improved the fit.

The described model was used to predict AmE, defined as the proportion of individuals with falciparum malaria infection effectively treated with the anti-malarial drug at different places and periods in the context of the actual clinical setting, while accounting for the health system, climate, environment, and socio-demographic factors. Models were fitted in a Bayesian framework using R-INLA [78, 79]. The Bayesian model formulation was completed by assigning a prior distribution to all unknown parameters and hyperparameters. The initial model assumed non-informative priors [78] and was further refined to include weakly informative priors if doing so improved model performance.

### Model validation and selection

A sensitivity analysis was done to examine the choice of priors for the hyperparameters, the likelihood of the outcome and inclusion of space–time interaction. For the temporal component, i.i.d., first-order random walk (RW1), second-order random walk (RW2), and first-order autoregressive (AR1) models were tested [80]. For the spatial components, range and variance, the effect of a non-informative prior was compared to penalized complexity (PC) priors [81], and a fixed range. Both beta and binomial distributions were considered for the outcome variable. Furthermore, a model with an overall temporal trend common to all spatial units and an overall spatial effect common to all time periods was compared against a model that allowed variation over space and time. Watanabe Akaike information criterion (WAIC), root mean squared errors (RMSE), and the correlation between the observed data and the predicted values, the R^2^, were used to compare the model fits. Using the variables retained from the first selection process, the space–time geostatistical model was built sequentially, starting with Model 1 containing no covariates; Model 2 with national level covariates; Model 3 with environmental and climate covariates; and, Model 4 with a combination of environmental, climate and national level covariates. All models included an annual time variable. Models were subjected to validation and calibration procedures using conditional predictive ordinate (CPO) and probability integral transform (PIT) to assess predictive performance [82–87].

Additional out-of-sample prediction was performed using a threefold cross-validation (~ 30/70 rule) in which the response data were split randomly into 3 non-overlapping subsets. Two sub-sets were used for model training, while the third sub-set was used for validation. This process was repeated 12 times for 4 different splits of the data. Through this method each observation was used in a test and training set. RMSE and R^2^, calculated between the observed and predicted data are used to assess model performance. Furthermore, in-sample and out-of-sample estimates of the RMSE were compared to assess over- or under-fitting. The statistics for model comparisons, together with results of validation/calibration and predictive ability, were used to select the best model. The models were fitted using 1991–2016 data. To forecast AmE for 2017, 2018 and 2019, the spatial pattern was assumed to be constant after 2016 and thus the pixel-level temporal trends in AmE were interpolated linearly from the time series models. The modelling framework is further explained in Additional file 1: Section 3—Figure S3.1.

### Calculation of final AmE estimates and presentation of results

The estimates obtained from the model were then adjusted for drug quality and patients’ adherence. Due to very limited available information on these important factors, uniform values were used for all regions and all time periods. First, based on the review by Ozawa et al., a penalty of 19.1% was applied to account for the prevalence of sub-standard or falsified anti-malarials [88]. Then a 5.0% penalization was used to account for the imperfect adherence to treatment [66]. To present the spatial distribution, the years between 1991 and 2019 were categorized to 1991–2000, 2001–2005, 2006–2010, 2011–2015, and 2016–2019. The last period is considered a forecasted period. The year ranges used in this categorization were selected based on temporal clustering of clinical efficacy trials and benchmark years for anti-malarial drug usage, including the WHO’s recommendation on ACT in 2001, peak of malaria burden in 2005, and high degree of adoption of ACT as first-line treatment in sub-Saharan Africa in 2011. Non-artemisinin-based anti-malarial effectiveness was not considered for the period after 2015 due to few non-artemisinin clinical efficacy trials being conducted after 2010. The resulting maps present the average AmE levels for the stated periods. Median and interquartile ranges (IQR) are used to report due to the asymmetric distribution of the AmE estimates. To capture uncertainty and spatial heterogeneity in the predicted AmE levels, stochastic realisations from the models’ posterior distributions were generated and then summarized to obtain mean and uncertainty estimates for national and sub-national administrative units.

## Results

### Study characteristics

The observed data covered the period 1991–2016. Trials on artemisinin-based anti-malarials (ACT + monotherapy) accounted for 88% of the studies included in this analysis. Most studies were performed in Africa (69.4%) and the most commonly tested anti-malarial was artemether–lumefantrine (n = 169) (Table 1). Among countries, Thailand (28 studies, 73 treatments), India (6 studies, 45 treatments), and Uganda (21 studies, 44 treatments) had the most studies on artemisinin-based anti-malarials. Uganda (32 treatments) and Madagascar (14 treatments) had the most studies on non-artemisinin-based anti-malarials (Additional file 1: Section 2). Descriptive analysis of all the treatments indicated that efficacy of artemisinin-based anti-malarial drugs remained high in most regions throughout the study period, while that of non-artemisinin-based declined over time. The median levels for artemisinin-based drugs obtained from the trials for the periods were 99.3% (1991–2000), 97.1% (2001–2005), 98.2% (2006–2010), and 98.8% (2011–2016). For the non-artemisinin drugs, these were 83.2% (1991–2000), 81.5% (2001–2005), 88.7% (2006–2010), and 51.6% (2011–2016).

### Model selection

The final specification of the model, with binomial likelihood and variation over space and time, used non-informative priors for the regression coefficients, PC priors for spatial effects and an AR1 structure for the temporal effects. The in-sample validation and model calibration indicated good performance of the models. Similar findings were obtained for the threefold cross-validation. The RMSE of the training set did not differ much from that of the validation set indicating that over-fitting was not an issue. The R^2^ between observed and predicted ranged between 0.86 and 0.89 (artemisinin-based) and 0.63 and 0.66 (non-artemisinin), depending on the set of covariates used. Model 4 had the best fit, as indicated by the lowest WAIC, and high predictive ability as indicated by high R^2^ (Table 2). Performance metrics for the final geostatistical models fitted for both drug types are shown in Table 2.

**Table 2:** Model assessment and selection in estimation of anti-malarial drug effectiveness

Metric	Non- artemisinin anti-malarial drugs				Artemisinin-based anti-malarial drugs			
	Model 1	Model 2	Model 3	Model 4	Model 1	Model 2	Model 3	Model 4
WAIC	3897.03	3760.59	3757.10	3749.13	4898.14	4855.83	4838.53	4800.72
RMSE	0.171	0.167	0.165	0.165	0.039	0.038	0.037	0.037
R-squared	0.635	0.654	0.664	0.665	0.86	0.87	0.88	0.89

Model 1—no covariates; Model 2—national level covariates; Model 3—environmental and climate covariates; Model 4—a combination of environmental, climate and national level covariates

*WAIC* Watanabe Akaike information criterion, *RMSE* root mean squared errors

### Anti-malarial drug effectiveness

**Fig. 1:**
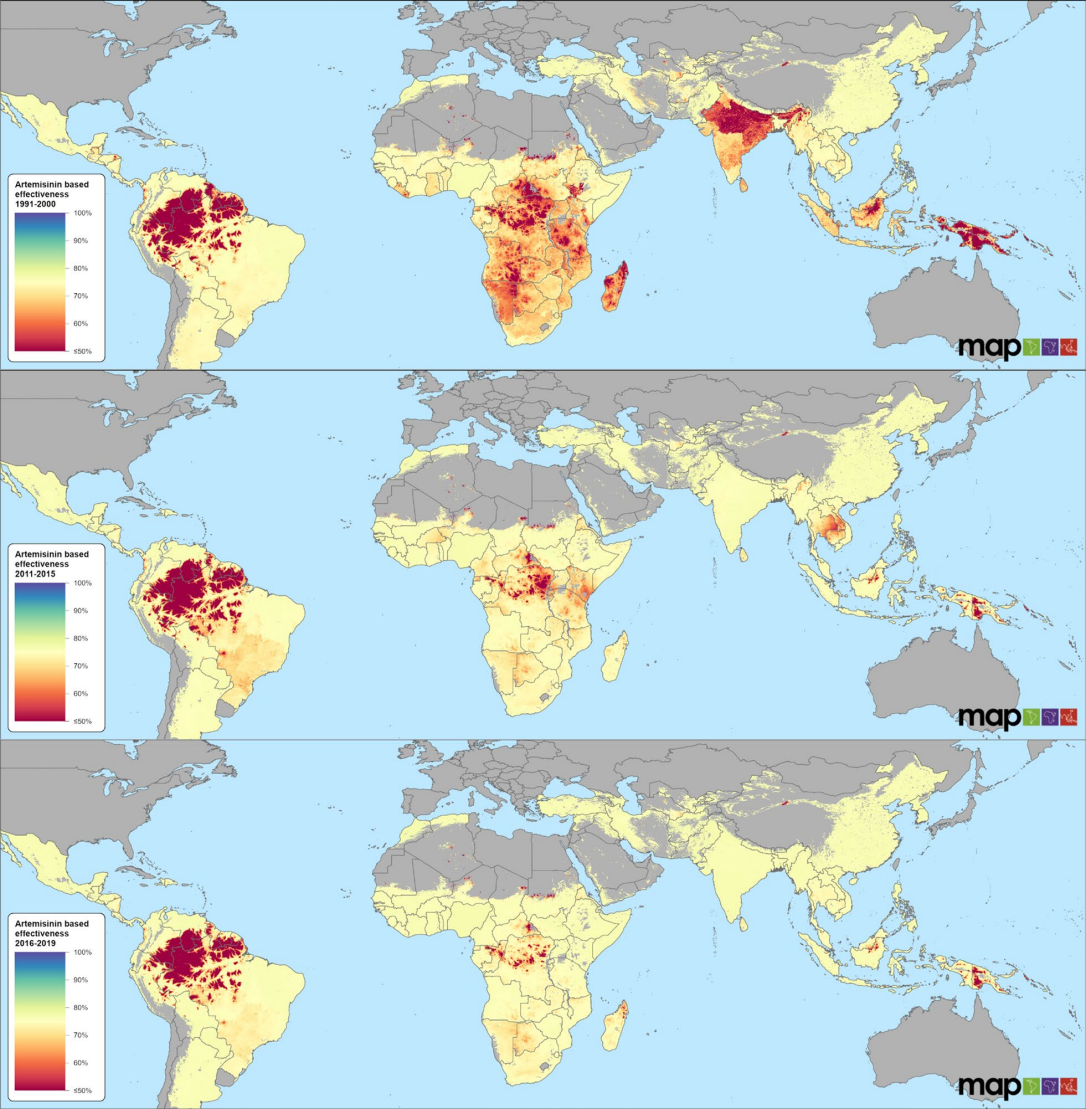
Spatiotemporal distribution of effectiveness of artemisinin-based anti-malarial drugs for periods 1991–2000, 2011–2015, and 2016–2019. Maps for 2001–2005 and 2006–2010 are presented in Additional file 1: Figure S4.1

**Fig. 2:**
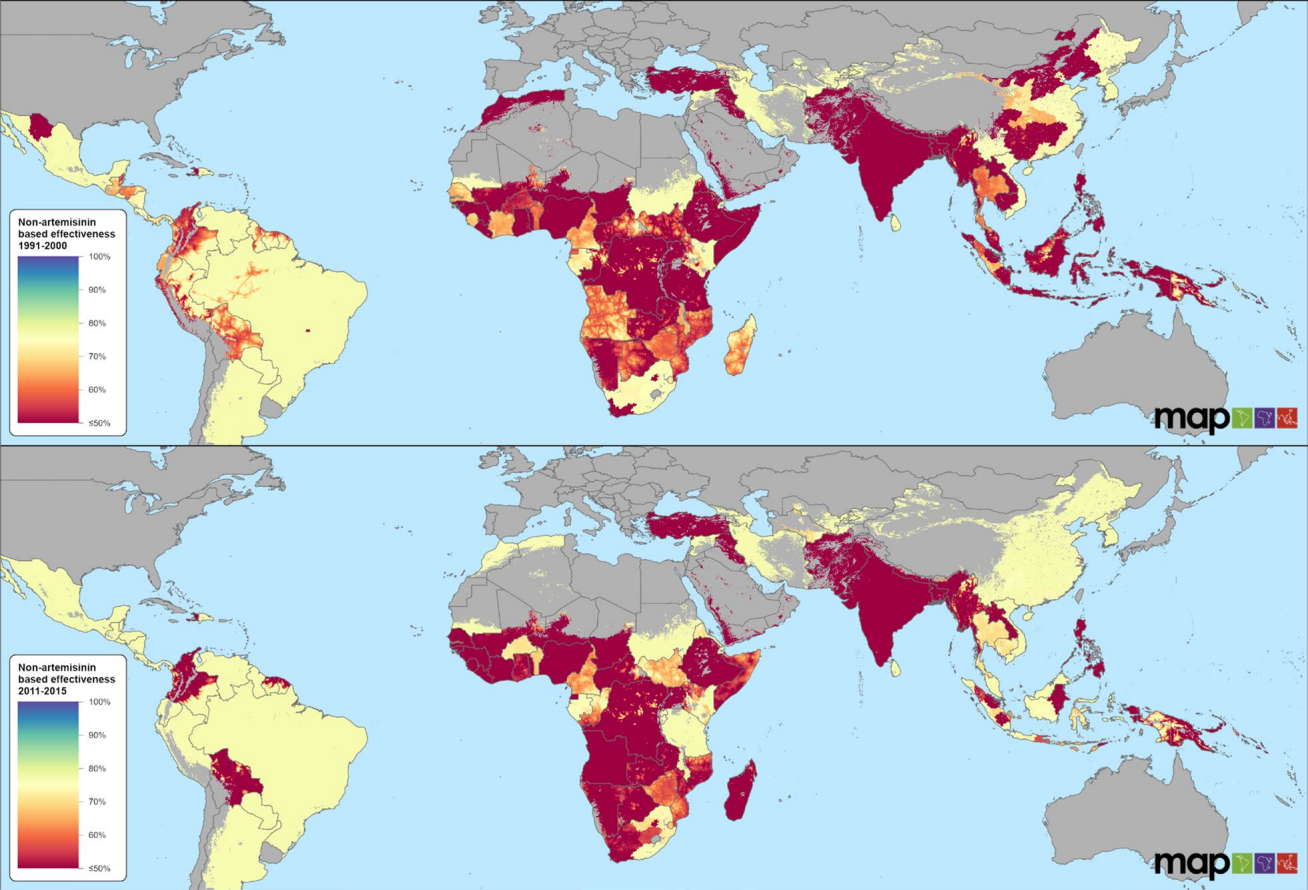
Spatiotemporal distribution of effectiveness of non-artemisinin anti-malarial drugs for periods 1991–2000 and 2011–2015. Maps for 2001–2005 and 2006–2010 are presented in Additional file 1: Figure S4.2

The AmE of artemisinin-based and non-artemisinin anti-malarial drugs was predicted annually for the period 1991–2019 for all malaria-endemic countries then aggregated into multi-year categories as indicated above. High-resolution maps (5 × 5 km) of AmE, adjusted for the drug quality and adherence, show the global spatial distribution and country-to-country variations (Figs. 1 and 2). Maps of the corresponding uncertainty measure, the IQR, are shown in Additional file 1: Section 4.

### Artemisinin-based anti-malarial drugs

The global AmE of artemisinin-based drugs was 67.4% (IQR: 33.3–75.8), 68.8% (IQR: 39.2–75.8), 70.1% (IQR: 43.6–76.0), 70.7% (IQR: 45.5–76.2), and 71.8% (IQR: 46.9–76.4) for the 1991–2000, 2001–2005, 2006–2010, 2011–2015, and 2016–2019 periods, respectively. Modelled AmE was 29.6% lower, on average, than the levels obtained from trials, largely due to the adjustments applied for drug quality and patient adherence. The relatively stable estimates over time indicated that AmE of artemisinin-based drugs remained high and increased by 6.5% from the years before 2000 to those after 2016 (Fig. 1). In the years before 2000, countries in sub-Saharan Africa, South America, India, and on the islands of the Indonesian archipelago were predicted to have lower AmE levels (< 60%) (Fig. 1). The situation improved substantially by the 2011–2015 period, with few remaining pockets of low AmE in sub-Saharan Africa. The levels of AmE dropped in Southeast Asian countries, including Thailand, Myanmar, Cambodia, and Laos. Little improvement in AmE was predicted for *P. falciparum*-endemic countries in South America including Guyana, Suriname, and parts of Brazil, Venezuela and Colombia. Generally, the model predicts improved AmE for artemisinin-based drugs for the period 2016–2019 (Fig. 1).

AmE levels often remained stable in countries where multiple ACT medicine was used as the first-line drug of choice, a practice which became more widespread in the mid-2000s. Noteworthy examples include Ghana, Nigeria, Senegal, Angola, and India. Factors positively related to ACT effectiveness in the analysis include access to health care, human resource capacity (skilled birth attendants as a proxy), education levels, and accessibility to cities. In contrast, AmE dropped in countries including Indonesia, Thailand and Vietnam where Dihydroartemisinin piperaquine (DHAP) was the sole first-line treatment and resistance emerged to either artesunate or the partner drug (Fig. 1).

### Non-artemisinin-based anti-malarial drugs

The global AmE of non-artemisinin-based drugs was much lower than the AmE of artemisinin-based drugs, and these estimates also had higher associated levels of uncertainty. Predicted AmE levels were 52.3% (IQR: 17.9–74.9), 53.1% (IQR: 21.6–74.0), 55.9% (IQR: 22.6–73.5), and 55.5% (IQR: 27.1–73.4) for the 1991–2000, 2001–2005, 2006–2010, and 2011–2015 periods, respectively. Predictions for these drugs beyond 2015 were not made due to data limitations. Globally, the AmE levels indicated an increase of 6.1% from the 1991–2000 period to the 2011–2015 period (Additional file 1: Section 4—Figure S4.2). The predicted AmE levels were approximately 36% lower than the mean values of measured effectiveness in a controlled trial setting. For all periods, the AmE estimates were lowest in sub-Saharan Africa and in Asia with a few countries observed to regain slight increases in effectiveness after 2011 (Fig. 2). In contrast to patterns elsewhere, the AmE of non-artemisinin-based drugs in Central and South America remained moderate and stable (Fig. 2).

National level covariates strongly affected the predicted effectiveness of non-artemisinin-based anti-malarial drugs. Health system and infrastructure factors, including coverage of skilled human resources, universal coverage, health care access, and accessibility to cities, were associated with increasing effectiveness. Factors negatively correlated with effectiveness included high levels of out of pocket expenditure and urbanization.

## Discussion

This study is the first attempt to produce high-resolution maps of spatiotemporal patterns of AmE, while accounting for the prevalence of sub-standard and falsified anti-malarials and patient adherence in all malaria-endemic countries. The resulting temporal patterns highlight changes in AmE from 1991 to 2019 while the spatial patterns illustrate heterogeneity between and within countries. Effectiveness of anti-malarial drugs used for treatment has a direct link to the progress of impact indicators, such as rates of malaria incidence and mortality in the population, which makes knowledge of this metric essential to control programmes. These findings provide additional evidence on practical considerations for implementing malaria treatment policies to ensure adequate anti-malarial effectiveness, including highlighting the roles that drug quality, adherence and health system quality play in AmE. Despite high clinical efficacy levels obtained under controlled conditions, AmE in artemisinin-based drugs dropped by at least a third when applied in the routine care delivery system. The drop was higher for the non-artemisinin-based drugs, likely due to increased level of resistance to those treatments. Overall, the results show that artemisinin-based anti-malarials have higher and more stable AmE compared to non-artemisinin-based drugs. For artemisinin-based anti-malarials, areas of lower AmE include the central and eastern part of sub-Saharan Africa, remote areas of South America, and Southeast Asia. The findings from this analysis suggest that non-artemisinin drugs remained effective for uncomplicated falciparum malaria in the South and Central American regions through 2015, but performed poorly elsewhere. However, this finding is associated with high levels of uncertainty due to little clinical efficacy data within the Americas. As such, this result should be viewed cautiously, and careful monitoring of both anti-malarial drug efficacy and health system performance metrics associated with effectiveness should accompany the continued use of non-ACT as front-line treatment in Central American countries. Resistance to non-artemisinin-based drugs has been observed in Africa since the 1980s [89] and inspired treatment policy changes to ACT since 2003 [90]. The WHO critical threshold for clinical efficacy is set at > 90%, but no documented threshold is set for AmE. Further investigation is needed in areas with low predicted effectiveness levels to determine factors driving the gap between the two metrics. Nevertheless, these findings suggest that, despite the availability of efficacious anti-malarials and over 80% of endemic countries adopting them as first line treatments, policy implementation gaps and challenges remain and impact malaria incidence and mortality. The emergence of resistance of artemisinin derivatives in Southeast Asia and its possible extension in other endemic regions may very negatively impact AmE, repeating patterns observed for non-artemisinin-based drugs in the past.

Time was an important parameter for modelling patterns of artemisinin-based AmE. In the early 2000s ACT was introduced, proved to be highly effective at treating malaria, and thus was adopted by many countries as a first-line drug to treat falciparum malaria. Prior to this period, artemisinin-based monotherapy was widely used, but this approach has since fallen out of favour as it is believed to promote emergence of drug resistance [14, 16]. In some regions, despite ACT being adopted as first-line treatment policy, their implementation has faced a number of challenges. Demographic and health survey and malaria indicators survey and other literature have shown that health workers still prescribe other anti-malarials for a range of reasons including patient preference, provider perception on specific types of anti-malarials, ACT stock-outs, costs including those incurred when accessing care, and higher availability and access to non-recommended anti-malarials. These multifaceted drivers of AmE have been reported across Africa, including in Kenya [91], Cameroon [92], Democratic Republic of Congo (DRC) [93], and Madagascar, and have slowed progress towards increasing AmE and reducing malaria burden [94]. The long term use of single anti-malarial drugs results in high drug-based selective pressure, which has been proven to decrease parasite sensitivity [95], and could explain the decreasing AmE patterns observed in some settings. Some countries introduced multiple ACT as first-line treatment options, which appears to have maintained the high AmE of ACT by reducing drug pressure. These policies also provided treatment choices to patients, which may have increased adherence. Countries adopting this strategy include Angola, Brazil, Burkina Faso, Nigeria, Senegal, Togo, Sierra Leone, China, and Myanmar [12, 96, 97]; countries with low ACT AmE could potentially use this as a mechanism for improving treatment success.

With the exception of Central America, where non-artemisinin-based AmE was higher in 2011–2015 than in 1991–2000, non-artemisinin drugs had reducing effectiveness in most areas and their efficacy changed less over time. This result is supported by the continued use of chloroquine as first-line treatment in several Central American countries [11]. For example, in Belize, Costa Rica, and El Salvador, first-line falciparum malaria treatment consists of chloroquine combined with primaquine (one-day dose) [11]. Using non-artemisinin-based drugs in combination and short-term dosing requirements may have slowed the development of drug resistance, increase patient adherence, efficacy and effectiveness, and led to the patterns observed in the findings (Additional file 1: Section 1) [11]. However, these findings are somewhat speculative as very few datasets on performance of anti-malarial drugs were available from Central and South America, resulting in more uncertain estimates. An unexpected finding of this work was a resurgence in non-ACT AmE in places where they have been banned, such as in Malawi [98]. However, as very few clinical trials on non-ACT have been conducted since the widespread adoption of ACT, this finding is driven by model covariates (e.g., improvements in health systems) rather than response data, and should be interpreted cautiously. By aggregating all ACT within a single analysis, direct assessment of known AmE limitations related to the efficacy of the partner drug (e.g., Artesunate–sulfadoxine–pyrimethamine) were not possible. These limitations include administration aspects, such as duration of treatment and the number of tablets in the dose regimens of the partner drugs [99, 100]. This could be a possible explanation for the lower drug effectiveness observed in Djibouti, Ecuador, India, Pakistan, and Sudan. Some of these countries changed their treatment policies to other ACT, such as Artemether–lumefantrine or DHAP, to increase effectiveness. However, even the most recently developed ACT, DHAP, faces a threat of resistance in some parts of the Greater Mekong Region [101]. Current efforts to sustain effective treatment of falciparum malaria in such areas include introduction of the triple artemisinin-based combination therapy (TACT) [99]. Lack of data on drug resistance prevented this important parameter from being included within the AmE models.

Health system factors are associated with anti-malarial AmE. Access to health care and human resource capacity influence how and which drugs are prescribed and used. With low access to health care, a significant number of cases will not reach the health care system. Such cases will either not receive any treatment or obtain treatment through other sources, the latter of which may result in the use of a non-first-line treatment and failure to record the type, quality and dosage of the drug within official statistics. In areas where drug monitoring is not effective, irrational provision and unregulated use of anti-malarials might be high, including sub-optimal dosage and increased use of falsified, sub-standard, or non-recommended medicines, all of which may lead to low effectiveness. Health workers’ skills and compliance with treatment guidelines, along with drug availability, determine which drugs are provided to patients and whether the treatments are properly managed, both of which are linked to an effective response to drugs [102]. Socio-demographic factors, including education and accessibility to cities, may have effects similar to those of access to health care and the knowledge of both the patient and health care provider. Political and economic upheaval are also likely to impact treatment AmE. For example, since the mid-1990s, the DRC and Central Republic of Africa have experienced high levels of violence, population displacement, and destruction of infrastructure, including health facilities. These factors reduced access to care, increased rates of infection, and led to poorer management [103]. Similarly, outbreaks of Ebola virus and SARS-CoV-2 (COVID-19) have shown the ability to devastate or overwhelm health care systems, which may disrupt access to core malaria interventions [41, 104]. The poor state of the health care systems within low-resource settings could explain low estimates of AmE in areas facing political instability despite the adoption of ACT as first-line treatment in these locations. In the Greater Mekong Region, malaria transmission patterns are rapidly evolving and there is vast spatial heterogeneity. International borders where transmission remains high are of particular concern, as these areas have poorer access to health care facilities and malaria surveillance measures [105]. Furthermore, malaria control measures are very hard to establish and implement effectively within highly mobile migrant populations [106–109].

This analysis has several noteworthy limitations that should be considered when evaluating estimates. First, the sparsity of efficacy trial data, particularly outside sub-Saharan Africa, led to high levels of uncertainty within the AmE result. This outcome stems from the challenges of conducting efficacy trials, which are costly in terms of money, time and effort, and are rarely conducted in regions with no evidence of treatment failure. Results of efficacy trials conducted by countries or the WHO are not systematically published or made available to stakeholders, nor is individual patient data shared which limits standardization of drug efficacy outcomes. Regional circumstances that are known to affect anti-malarial intervention programmes may also prevent clinical trials from being conducted (e.g., chronic warfare has hampered the implementation of malaria control interventions in South Sudan [110]). To mitigate these important data limitations, countries are urged to explore potential mechanisms to utilize routine surveillance systems for continuous assessment of anti-malarial drugs performance. Future analysis utilizing these estimates may benefit from including country-specific primary data related to utilization of health services and anti-malarials. Routine data from countries provides opportunities to refine this estimate iteratively. A second noteworthy limitation is that this study did not utilize patient-level or pharmacokinetic/pharmacodynamic (PK/PD) data. Such data would provide patient and drug information (e.g., parasitaemia levels, status of fever, genetics, drug concentration, and biological processes) that could be used to refine estimates [111]. Third, individuals are included in clinical trials only if they meet eligibility criteria, which may result in under-representation of the segments of the population most affected by malaria. This could occur, for example, if clinical trials over-represent adults when children under five years represent the majority of cases. As a result, trial results may not be generalizable to the real-world population. Fourth, due to non-availability of data on adherence and quality of anti-malarials medicine used for treatment, a constant adjustment was applied uniformly over space and time. These metrics are likely to vary, therefore this scaling may over- or underestimate AmE. Fifth, the socio-demographic covariates used in this analysis were modelled using a limited set of predictor variables and are somewhat collinear despite a variable selection process conducted. This could hinder the interpretation of results by producing circularity within downstream assessments of causal relationships between AmE and metrics of national development. While this is worth noting, this is not a critical limitation to the study, but should be considered if these results are used in an analysis with other GBD covariates. Sixth, most of the covariates are modelled products that are associated with uncertainty, which is difficult to fully propagate within modelling frameworks. The noise inherited in the efficacy estimates from the WWARN database is expected to be the largest contributor of uncertainty in the AmE estimates, as suggested by the random effects, and thus uncertainty in the covariates was comparatively minor but could still lead to underestimated uncertainty in the final results. Finally, the uncertainty related to AmE estimates varies across space and time, which is characterized by producing multiple realisations of AmE for each year. By summarizing these realisations, mean and uncertainty maps are produced and made available for download so that other researchers can propagate these uncertainties through their analyses appropriately.

## Conclusions

This work utilized data from clinical efficacy trials to produce global-scale predictions of anti-malarial drug effectiveness, while also incorporating information on drug quality and patient adherence. Triangulating modelling and policy decision thresholds, these predictions provide new insights and help characterize spatial patterns in effectiveness, which provide evidence-based and geographically explicit guidelines for optimal medication-based malaria control worldwide. Most malaria-endemic countries are progressing towards elimination, with anti-malarial drugs playing a key role in driving down malaria burden. Prompt diagnosis and effective treatment of cases remain an important strategy in the management and control of malaria in all endemic countries. As such, understanding the state of anti-malarial drug effectiveness becomes a crucial component. Findings from this study provide evidence that ACT remains highly effective as first-line treatment for uncomplicated falciparum malaria compared to non-artemisinin based anti-malarials. However, the drug efficacy levels reported in clinical settings are unlikely to reflect real-world efficacy. Results obtained from this analysis suggest that strategies used in different countries to sustain the effectiveness of these drugs are working, including the use of multiple options for first-line treatment and combining artemisinin with other anti-malarial drugs that remain effective locally. These findings are relevant for guiding policy decisions on targeted interventions towards malaria and contribute to global malaria burden estimates in the World Malaria Report and the GBD study.

## Supplementary information

**Additional file 1.** Supplementary information on methods, data and findings.

## Data Availability

The datasets used and/or analysed during the current study are available from the corresponding author on reasonable request.

## Abbreviations

ACT: Artemisinin-based combination therapy
AL: Artemether–lumefantrine
AmE: Anti-malarial drug effectiveness
ASAQ: Artesunate–amodiaquine
ASMQ: Artesunate–mefloquine
ASSP: Artesunate–sulfadoxine–pyrimethamine
DHAP: Dihydroartemisinin–piperaquine
GBD: Global burden of disease
IHME: Institute for Health Metrics and Evaluation
IQR: Interquartile range
MAP: Malaria Atlas Project
PC: Penalized complexity
WHO: World Health Organization
WWARN: Worldwide Anti-malarial Resistance Network
