# Supplementary material for: Global estimation of anti-malarial drug effectiveness for the treatment of uncomplicated Plasmodium falciparum malaria 1991–2019
Source: Malar J. 2020 Oct 20;19:374. doi: 10.1186/s12936-020-03446-8 (PMC7573874; doi:10.1186/s12936-020-03446-8)
Supplement: Supplementary file 1 — Additional file 1. Supplementary information on methods, data and findings. [file 12936_2020_3446_MOESM1_ESM.docx]

**Additional File**

**Global estimation of antimalarial drug effectiveness for the treatment of uncomplicated *Plasmodium falciparum* malaria 1991 – 2019**

*Corresponding author: susan.rumisha@bdi.ox.ac.uk*

**Section 1: Definition of therapeutic efficacy study endpoints, inclusion criteria, useful terms by WHO and WWARN and First-line treatment for malaria in endemic countries by WHO Malaria Report**

**Table S1.1: Therapeutic efficacy study endpoints [1]**

| **End-point for day X**  **(X = 28 or 42)** | **Cumulative success or failure rate (Kaplan-Meier analysis)** | **Proportion**  **(per-protocol analysis)** |
| --- | --- | --- |
| Adequate clinical and parasitological response at day X | Success | Success |
| Early treatment failure | Failure | Failure |
| Late clinical failure before day 7 | Failure | Failure |
| Late clinical failure or late parasitological failure on or after day 7 |  |  |
| - *P. falciparum* recrudescence^[[1]](#footnote-1)^* | Failure | Failure |
| - *P. falciparum* reinfection^[[2]](#footnote-2)^* | Censored day of reinfection | Excluded from analysis |
| - other species with  *P. falciparum* recrudescence | Failure | Failure |
| - other species with  *P. falciparum* reinfection | Censored day of reinfection | Excluded from analysis |
| - other species infection | Censored day of infection | Excluded from analysis |
| - undetermined or missing PCR | Excluded from PCR-corrected analysis but included as failures for PCR-uncorrected analysis | Excluded from analysis |
| Loss to follow-up | Censored last day of follow-up according to timetable | Excluded from analysis |
| Withdrawal and protocol violation | Censored last day of follow-up according to timetable before withdrawal or protocol violation | Excluded from analysis |

In the PCR adjusted analysis, treatment failures were defined as:

- Early treatment failures
- *P. falciparum* parasitaemia between day 4 and 7 after treatment unless PCR results indicated reinfection
- Late treatment failure (LCF or LPF) after day 7 with *P. falciparum* parasitaemia (alone or mixed) with PCR-confirmation of recrudescence

Censoring occurred when patients had recurrent parasitaemia after day 7 where no PCR confirmation of recrudescence or reinfection was obtained.

In the PCR unadjusted analysis, treatment failures were defined as:

- Early treatment failures
- Patients with recurrent *P. falciparum* parasitaemia after day 4 until end of follow-up will be censored at the day of the occurrence.

The WHO’s protocol for monitoring antimalarial drug efficacy relies on efficacy trials including therapeutic efficacy studies (TES). TES utilize standardized protocols, implementation, and analysis, and are thus considered the gold standard for monitoring antimalarial drug efficacy. Because they are standardized, TES provide a mechanism for conducting cross-comparisons between different trials, comparing results within and between countries, and assessing efficacy over time. Furthermore, TES allow the detection of subtle changes in treatment outcomes and provide critical inputs used by national malaria control programs (NMCPs) to decide on treatment policies. However, the WHO protocols for conducting TES have been revised several times to alter the mandatory duration of follow-up and the definition of primary endpoints. These changes make comparisons between recent and historical data challenging. Likewise, data from efficacy studies may be difficult to compare as they are analysed using ‘per protocol’, ‘intention to treat’, or ‘modified intention to treat’ methodologies. These methodologies differ in how they exclude patient data based on their adherence to the protocol and clinical outcomes, such as loss to follow-up, late clinical and parasitological failures, recurrent parasitaemia, or new falciparum infections. Simple re-analysis may introduce biases when using data combined from studies where patients were followed-up at different periods, conducted in different malaria transmission zones, or analysed using different statistical methods. Such exclusion criteria and biases can result in incorrect estimates of efficacy levels and thus misguide policy decisions.

**WWARN and WHO therapeutic efficacy study inclusion criteria [2]**

- age, 6-59 months, i.e. under 5 years in areas of high transmission, and all patients over 6 months of age in areas of low-to-moderate transmission;
- mono-infection with *P. falciparum* detected by microscopy;
- asexual parasite count of 2000 – 200 000/ µl in areas of high transmission and 1000 – 100 000/ µl in areas of low-to-moderate transmission;
- axillary temperature ≥ 37.5°C or history of fever during the 24 h before recruitment;
- ability to swallow oral medication;
- ability and willingness to comply with the protocol for the duration of the study and to comply with the study visit schedule;
- informed consent from the patient or from a parent or guardian in the case of children
- absence of general danger signs in children under 5 years or signs of severe *P. falciparum* malaria according to the definitions of WHO [3];
- absence of severe malnutrition according to WHO child growth standards [4];
- absence of febrile condition due to diseases other than malaria (e.g. measles, acute lower respiratory tract infection, severe diarrhoea with dehydration) or other known underlying chronic or severe diseases (e.g. cardiac, renal or hepatic diseases, HIV/ AIDS);
- absence of regular medication, which might interfere with antimalarial pharmacokinetics;
- absence of history of hypersensitivity reactions or contraindication to any medicine being tested or used as alternative treatment; and
- a negative pregnancy test or not breastfeeding.

**Definition of terms (5, 6)**

*Treatment failure*

Inability to clear malarial parasitaemia or resolve clinical symptoms despite administration of an antimalarial medicine.

*Drug Efficacy*

Performance of a drug under ideal and controlled setting.

*Drug Effectiveness*

Performance of a drug under 'real-world' conditions. The usual setting of healthcare practice where patient populations and other variables cannot be controlled.

*Drug resistance*

The ability of a parasite strain to survive or multiply despite the administration and absorption of a drug given in doses equal to or higher than those usually recommended but within the tolerance of the subject. The form of the drug active against the parasite must be able to gain access to the parasite or the infected erythrocyte for the duration of the time necessary for its normal action.

*Per-protocol analysis*

Analysis of clinical trial data which include only those patients who complete the entire study follow-up and have a clear outcome of either treatment success or failure. Patients who do not complete follow-up, deviate from the study protocol or withdraw are excluded entirely.

*Intention-to-treat*

Analysis of clinical trial data which include all patients. The outcomes of all patients are designated as either a success or a failure. Patients who do not complete the study are generally classified as having treatment failure.

Brief note on existing databases of drug efficacy trials or TES

WHO database focused on documenting treatment policy changes in malaria-endemic countries. The interdisciplinary monitoring project for antimalarial combination therapy (IMPACT) programme, which was conducted in Tanzania, prioritised assessing the efficacy of artesunate-sulfadoxine-pyrimethamine (ASSP) only. Other related databases include those from regional networks, including the East African network for monitoring antimalarial treatment (EANMAT), the Mekong Network, which focuses on Southeast Asia and South America; and the Institute Pasteur international network which focuses on Cambodia.

Table S1.2: First line treatment for malaria endemic countries. The data were compiled from World Malaria Reports and indicated the change of policy from 2001 to 2016 (7-9).

| **Country** | **First-line treatment**  **2001 - 2005** | **First-line treatment**  **2006 - 2010** | **First-line treatment**  **2011 - 2016** |
| --- | --- | --- | --- |
| Afghanistan | ASSP | ASSP | ASSP + PQ |
| Algeria |  |  |  |
| Angola | CQ | AL | AL |
|  |  |  | ASAQ |
|  |  |  | DHAP |
| Argentina |  |  | AL + PQ |
| Armenia | CQ |  |  |
| Azerbaijan | CQ |  |  |
| Bangladesh | AL | AL | AL |
| Belize |  | CQ | CQ+ PQ |
| Benin | AL | AL | AL |
| Bhutan | AL | AL | AL |
| Bolivia | ASMQ | ASMQ | AL |
|  | CQ |  |  |
| Botswana | SP | AL | AL |
| Brazil | QN + PQ | AL | AL + PQ |
|  |  | ASMQ | ASMQ+PQ |
| Burkina Faso | CQ | AL | AL |
|  |  | ASAQ | ASAQ |
| Burundi | ASAQ | ASAQ | ASAQ |
| Cape Verde | CQ | AL | AL |
| Cambodia | ASMQ | ASMQ | ASMQ |
|  |  | DHAP + PQ |  |
| Cameroon | ASAQ | ASAQ | ASAQ |
| Central African Republic | CQ | AL | AL |
| Chad | CQ | AL | AL |
|  |  | ASAQ | ASAQ |
| China | AS | PQ | ASAQ |
|  | DHAP |  | DHAP |
| Colombia | SP | ASMQ | AL + PQ |
| Comoros | AL | AL | AL |
| Congo | CQ | ASAQ | ASAQ |
| Costa Rica |  | CQ+ PQ | CQ+ PQ |
| Cote d'Ivoire | SP | ASAQ | ASAQ |
| Democratic People's Republic of Korea |  |  |  |
| Democratic Republic of the Congo | SP | ASAQ | ASAQ |
| Djibouti | CQ | ASSP | AL + PQ |
| Dominican Republic | CQ+ PQ | CQ+ PQ | CQ+ PQ |
| Ecuador | ASSP | ASSP | AL + PQ |
| Egypt | CQ | AL |  |
| El Salvador |  | CQ+ PQ | CQ+ PQ |
| Equatorial Guinea | CQ | ASAQ | ASAQ |
| Eritrea | CQ | ASAQ | ASAQ |
|  | SP |  |  |
| Ethiopia | AL | AL | AL |
| French Guiana | QN | AL | AL |
| Gabon | ASAQ | ASAQ | ASAQ |
| Gambia | AL | AL | AL |
| Georgia | CQ |  |  |
| Ghana | ASAQ | AL | AL |
| Guatemala |  |  | CQ+ PQ |
| Guinea | CQ | ASAQ | ASAQ |
| Guinea-Bissau | CQ | AL | AL |
| Guyana |  | AL + PQ | AL + PQ |
| Haiti | CQ+ PQ | CQ+ PQ | CQ+ PQ |
| Honduras |  | CQ+ PQ | CQ+ PQ |
| India | ASSP | ASSP | ASSP+PQ |
|  | CQ |  | AL |
| Indonesia | ASAQ | DHAP + PQ | DHAP + PQ |
|  |  | ASAQ |  |
| Iran | ASSP | ASSP | ASSP + PQ |
| Iraq |  | AL |  |
| Kenya | AL | AL | AL |
| Kyrgyzstan | CQ |  |  |
| Laos | AL | AL | AL |
|  | CQ |  |  |
|  | SP |  |  |
| Liberia | ASAQ | ASAQ | ASAQ |
| Madagascar | ASAQ | ASAQ | ASAQ |
| Malawi | SP | AL | AL |
| Malaysia | CQ | ASMQ | ASMQ |
|  | SP |  |  |
| Maldives | CQ |  |  |
| Mali | AL | AL | AL |
|  |  | ASAQ | ASAQ |
| Mauritania | CQ | AL | AL |
|  |  | ASAQ | ASAQ |
| Mayotte |  |  | AL |
| Mexico |  | CQ+ PQ | CQ+ PQ |
| Morocco | AL |  |  |
| Mozambique | SP | AL | AL |
| Myanmar | AL | AL | AL |
|  | ASMQ | ASMQ | ASMQ |
|  |  | DHAP | DHAP |
|  |  |  | PQ  AM |
| Namibia | AL | AL | AL |
| Nepal | SP | AL | AL+PQ |
| Nicaragua |  | CQ+ PQ | CQ+ PQ |
| Niger | AL | AL | AL |
| Nigeria | AL | AL | AL |
|  |  | ASAQ | ASAQ |
| Oman | SP | AL + PQ |  |
| Pakistan | CQ | ASSP | ASSP + PQ |
| Panama |  | SP | AL + PQ |
| Papua New Guinea | CQ | AL | AL |
|  | SP |  |  |
| Paraguay | CQ+ PQ | CQ+ PQ | AL + PQ |
| Peru | ASMQ | ASMQ | ASMQ+PQ |
|  | ASSP |  |  |
| Philippines | SP | AL+PQ | AL+PQ |
|  | CQ |  |  |
| Republic of Korea |  |  |  |
| Rwanda | SP | AL | AL |
| Sao Tome and Principe | ASAQ | ASAQ | ASAQ |
| Saudi Arabia | CQ | ASSP | ASSP + PQ |
| Senegal | SP | AL | AL |
|  |  | ASAQ | ASAQ |
|  |  |  | DHAP |
| Sierra Leone | ASAQ | ASAQ | ASAQ |
|  |  |  | AL |
| Solomon Islands | CQ | AL | AL |
|  | SP |  |  |
| Somalia | CQ | ASSP | AL + PQ |
| South Africa | AL | AL | AL |
|  | ASSP |  | QN+CL |
|  |  |  | QN+D |
| South Sudan | ASAQ | ASAQ | ASAQ |
| Sri Lanka | CQ | AL + PQ |  |
| Sudan | ASSP | ASSP | AL |
| Suriname | AL | AL | AL + PQ |
| Swaziland | CQ | AL | AL |
| Syrian Arab Republic | SP | AL + PQ |  |
| Tajikistan | ASSP | AL |  |
| Tanzania | AL | AL | AL |
| Thailand | ASMQ | ASMQ | DHAP |
| Timor-Leste | SP | AL | AL+PQ |
| Togo | CQ | AL | AL |
|  |  | ASAQ | ASAQ |
| Turkey | CQ |  |  |
| Turkmenistan | CQ |  |  |
| Uganda | AL | AL | AL |
| United Arab Emirates | SP |  |  |
| Uzbekistan | CQ |  |  |
| Vanuatu | CQ | AL | AL |
| Vanuatu | SP |  |  |
| Venezuela | ASMQ | ASMQ+PQ | ASMQ+PQ |
| Vietnam | DHAP | DHAP | DHAP |
|  | AS |  |  |
| Yemen | CQ | ASSP | ASSP |
| Zambia | AL | AL | AL |
| Zanzibar | ASAQ | ASAQ | ASAQ |
| Zimbabwe | CQ | AL | AL |
|  | SP |  |  |

Key: AS = monotherapy artesunate; AML = artemether-lumefantrine; DHAP = dihydroartemisinin-piperaquine; ASAQ = artesunate-amodiaquine; ASMQ = artesunate-mefloquine; ASSP = artesunate–sulfadoxine–pyrimethamine; CQ = chloroquine; SP = sulfadoxine-pyrimethamine; QN + CL = Quinine plus clindamycin; QN+D = quinine plus doxycycline; PQ = Primaquine

**Section 2: Distribution of efficacy studies by treatment, continent, country and year**

**Table S2.1: Number of studies by treatment category for each country***

| Country | Non-artemisinin-based | Artemisinin-based |
| --- | --- | --- |
| Afghanistan | 1 | 2 |
| Angola | 2 | 5 |
| Bangladesh | 1 | 4 |
| Benin | 2 | 3 |
| Brazil |  | 2 |
| Burkina Faso | 7 | 14 |
| Cambodia |  | 6 |
| Cameroon |  | 3 |
| Central African Republic | 1 | 1 |
| Chad | 2 |  |
| China |  | 2 |
| Colombia | 2 | 2 |
| Congo |  | 4 |
| Cote d'Ivoire |  | 7 |
| Democratic Republic of the Congo | 2 | 6 |
| Eritrea |  | 1 |
| Ethiopia |  | 3 |
| Gabon | 2 | 5 |
| Ghana |  | 1 |
| Guinea |  | 1 |
| Guinea-Bissau | 3 | 2 |
| Guyana |  | 1 |
| India | 1 | 6 |
| Indonesia |  | 2 |
| Kenya | 2 | 14 |
| Laos | 2 | 5 |
| Liberia | 1 | 3 |
| Madagascar | 1 | 3 |
| Malawi | 1 | 3 |
| Mali | 2 | 9 |
| Mozambique | 4 | 5 |
| Myanmar | 1 | 5 |
| Niger |  | 3 |
| Nigeria |  | 3 |
| Papua New Guinea |  | 3 |
| Peru |  | 1 |
| Rwanda | 2 | 3 |
| Senegal | 1 | 11 |
| Sierra Leone | 4 | 1 |
| Somalia |  | 1 |
| South Africa | 2 | 2 |
| Sudan | 2 | 4 |
| Tanzania | 1 | 12 |
| Thailand | 12 | 28 |
| The Gambia | 2 | 3 |
| Togo |  | 1 |
| Uganda | 18 | 21 |
| Vietnam |  | 3 |
| Yemen |  | 1 |
| Zambia |  | 3 |

* Some studies included both treatments hence are counted twice

**Figures S2.1 – S2.2** show the distribution of studies by countries and the efficacy levels by type of drug studied for artemisinin and non-artemisinin categories. The cut-off point for clinical drug trials is 90% efficacy, the WHO states that medications below this level should be regarded non-efficacious in the area.


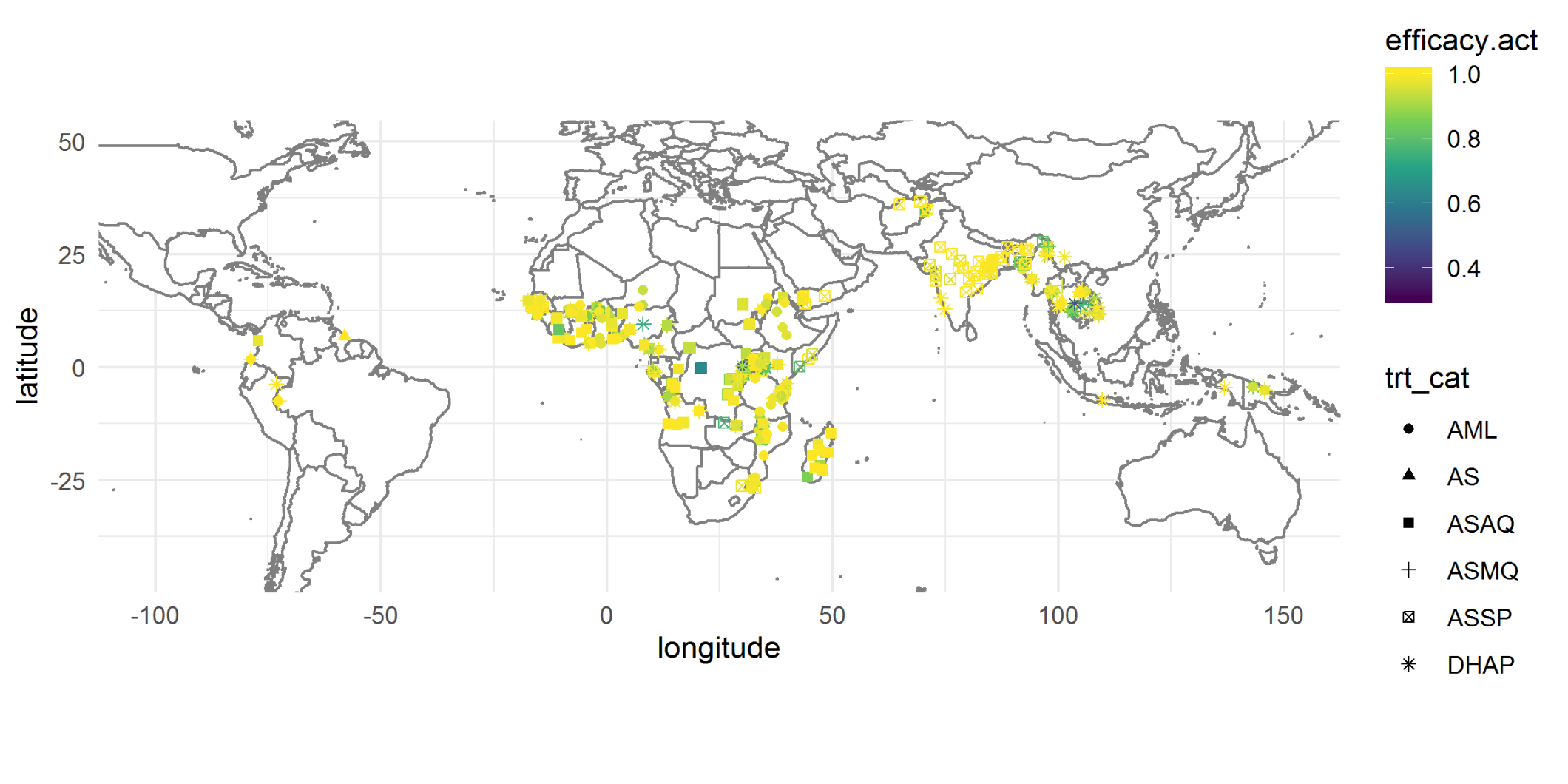


Figure S2.1 - Distribution of efficacy of artemisinin-based antimalarial based on therapeutic efficacy studies by country and treatment, 1991-2016; AS = monotherapy artesunate; AML = artemether-lumefantrine; DHAP = dihydroartemisinin-piperaquine; ASAQ = artesunate-amodiaquine; ASMQ = artesunate-mefloquine; ASSP = artesunate–sulfadoxine–pyrimethamine


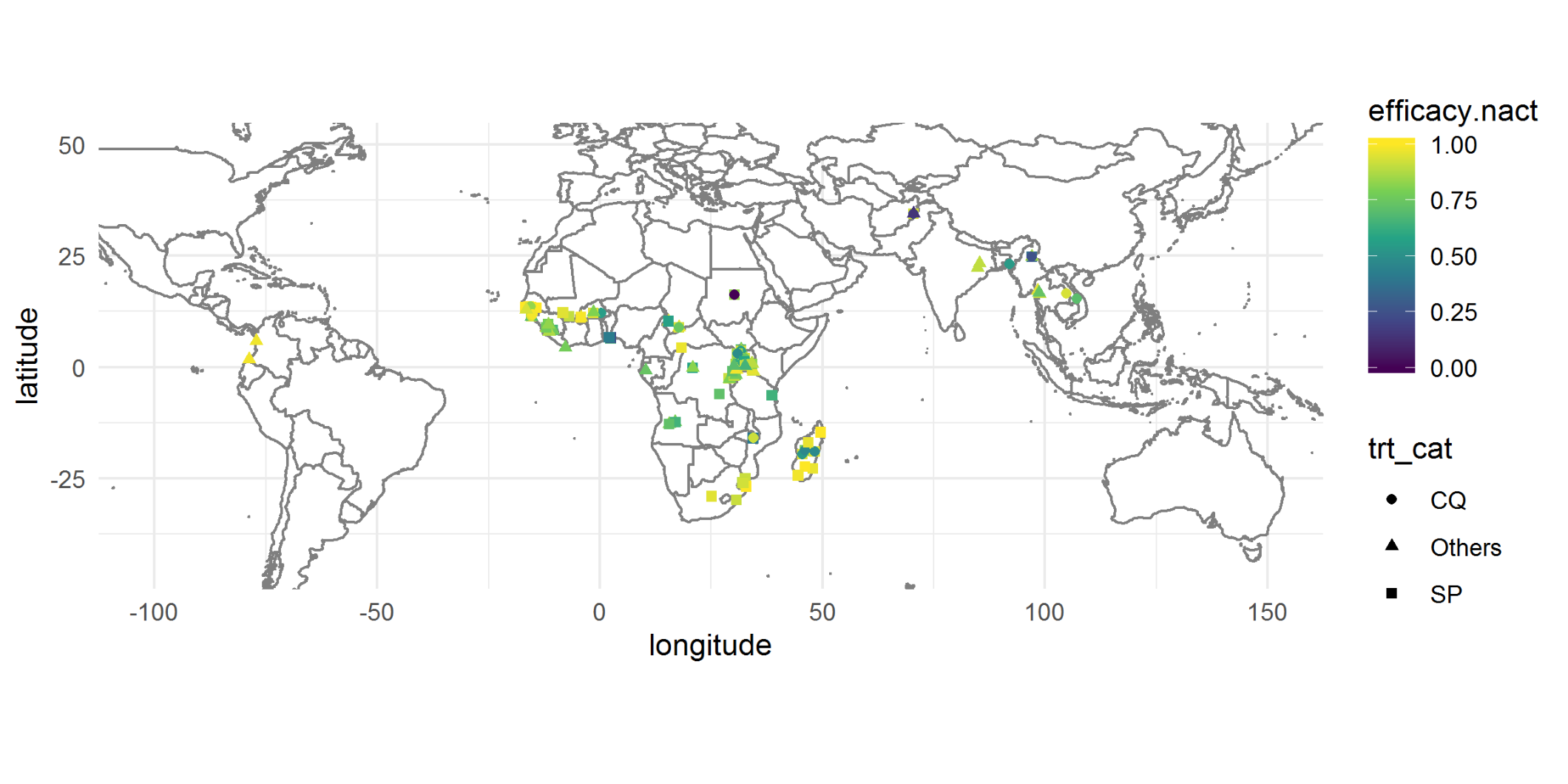


Figure S2.2 - Distribution of non-artemisinin therapeutic efficacy studies by country and treatment, 1991-2016; CQ = chloroquine; SP = sulfadoxine-pyrimethamine; Others = chlorproguanil-dapsone, halofantrine, quinine.

Efficacy level by continent and years

**Figures S2.3 – S2.4** show the median efficacy levels of artemisinin and non-artemisinin drugs by continent from observed trials, 1991-2016.

*
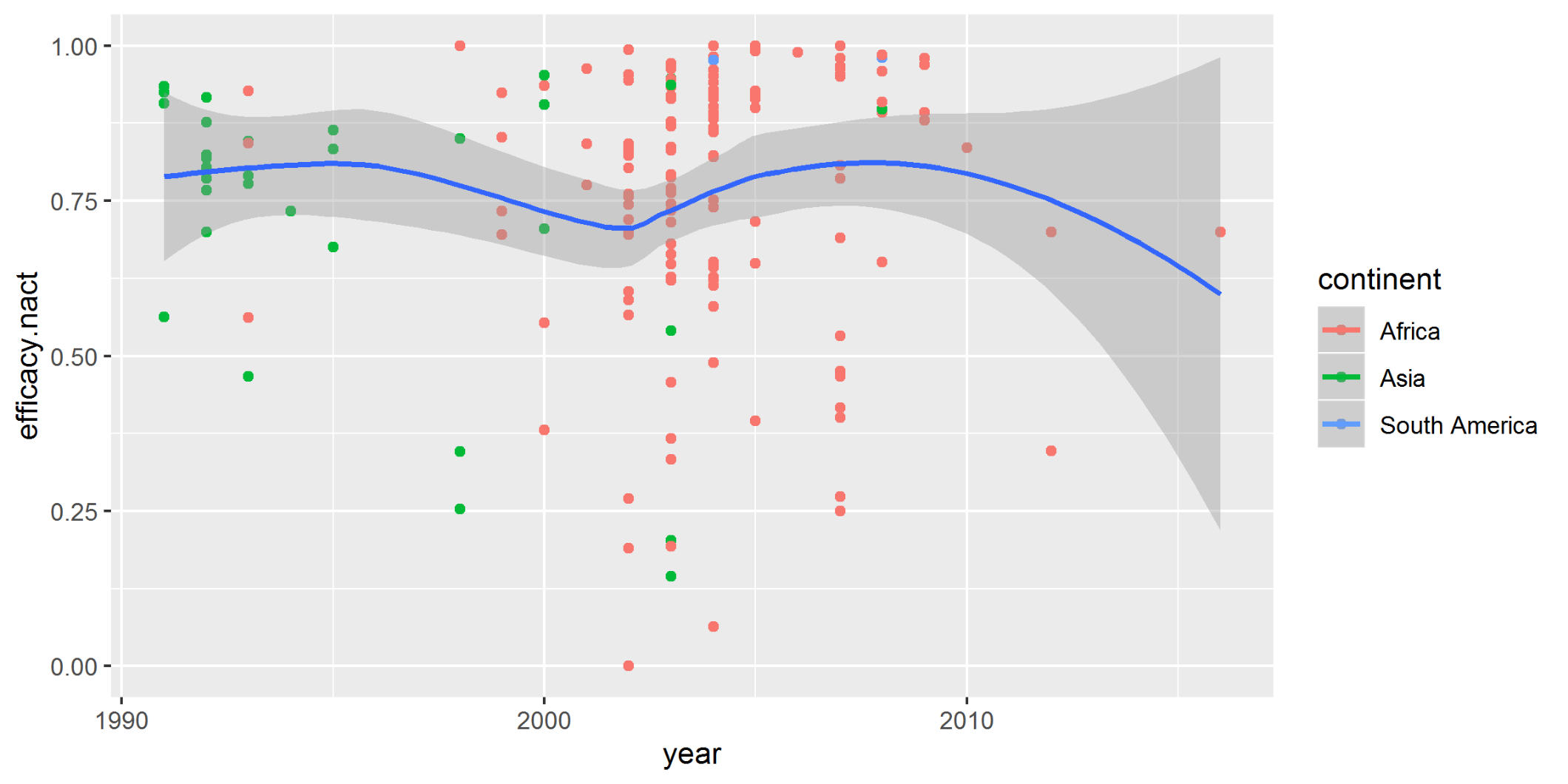
*

*Figure S2.3 - Efficacy levels of non-artemisinin-based drugs from observed trial data by continent, 1991-2016. The blue line indicates a smooth function while the grey shaded areas shows uncertainty level*

*
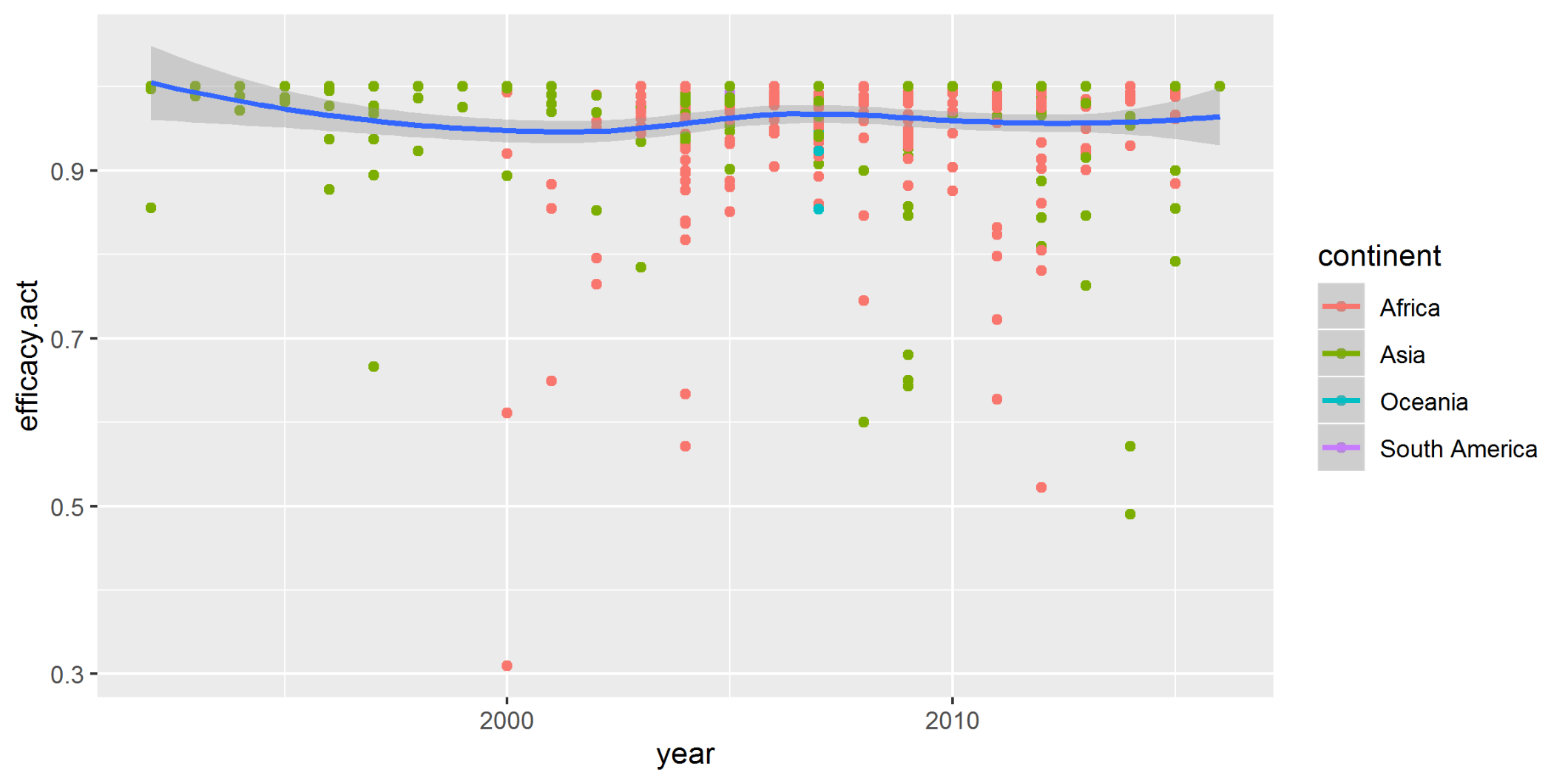
*

*Figure S2.4 - Efficacy levels of artemisinin-based drugs from observed trial data by continent, 1991-2016. The blue line indicates a smooth function while the grey shaded areas shows uncertainty level*

Efficacy by year period and country

**Figures S2.5 – S2.6** show the distribution of studies by countries and the efficacy levels for artemisinin and non-artemisinin categories in four different time periods. The cut-off point for clinical drug trials is 90% efficacy, the WHO states that medications below this level should be regarded non-efficacious in the area.

*
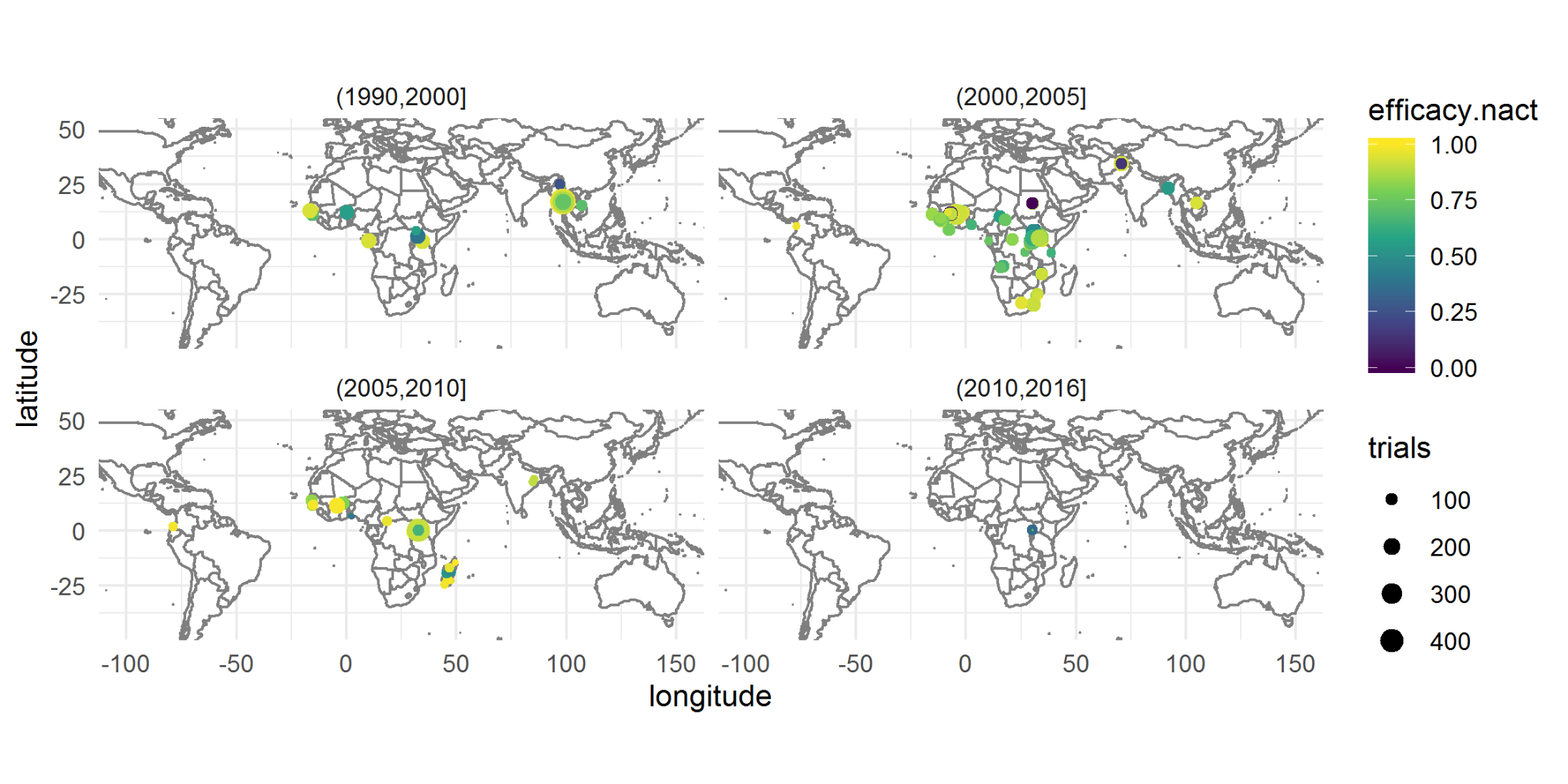
*

*Figures S2.5 – Distribution of studies by countries, years and the efficacy levels for non-artemisinin-based drugs*

*
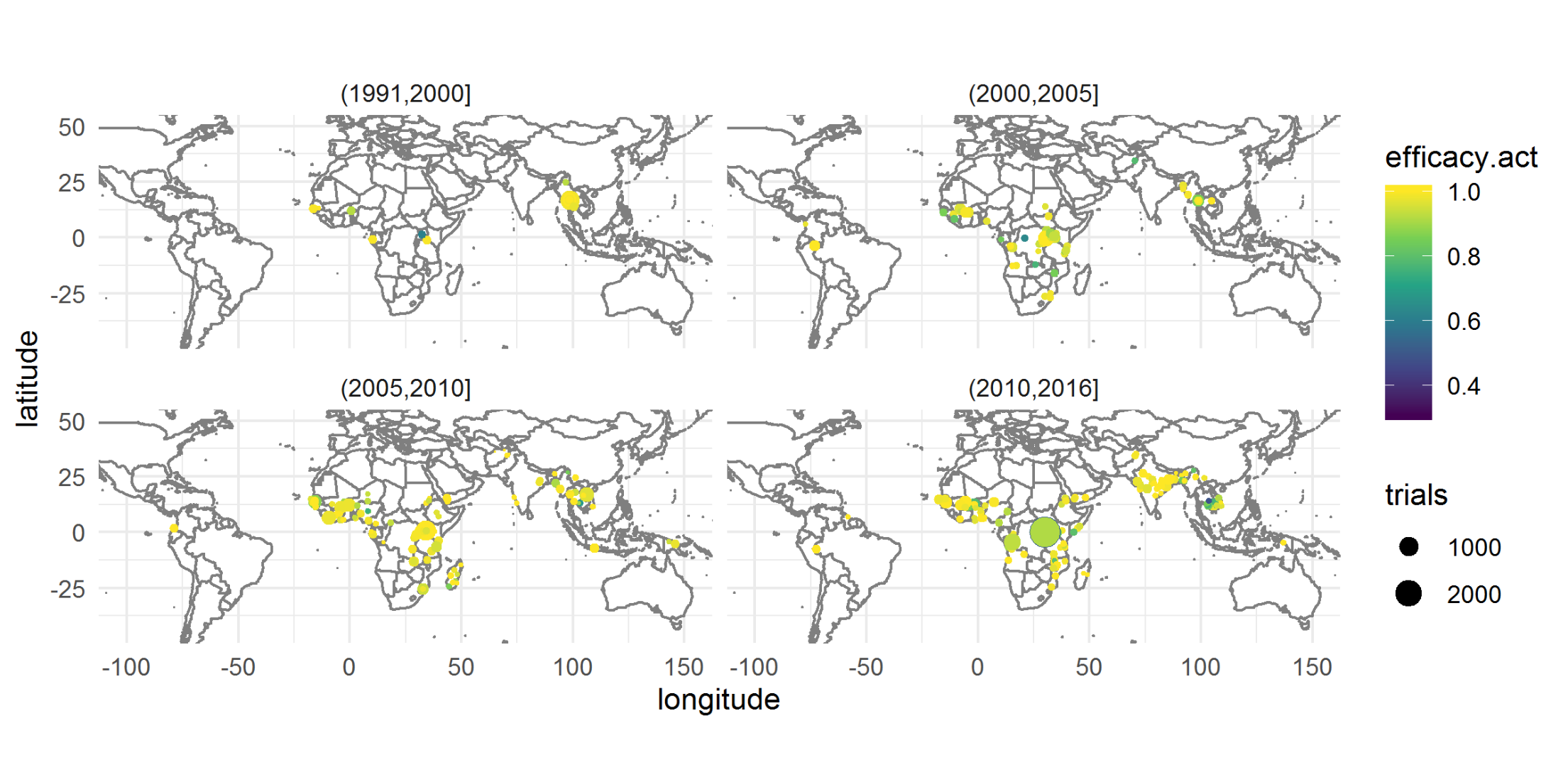
*

*Figures S2.6 – Distribution of studies by countries, years and the efficacy levels for artemisinin-based drugs*

**Section 3: Covariates considered for modelling antimalarial drug effectiveness**

List of covariates considered in the antimalarial effectiveness modelling. It includes variable identified from the literature review, those extracted from Institute of Health Metrics and Evaluation (IHME) and Malaria Atlas Project (MAP) databases.

The literature review: A critical review was done to identify relevant covariates to be considered for the analysis. Each of the literature in the WWARN database was screened manual and papers studied falciparum malaria, exclude pregnant women, and had a follow-up time of at least 28 days were included. Other literature were searched from multiple databases including PubMed, Embase, the Web of Science library and Google Scholar using relevant search terms. The search terms were developed to include all antimalarials used for treatment of falciparum malaria. Among the outcome searched was antimalarial drug efficacy, treatment failure, effectiveness of antimalarials, quality of antimalarials, antimalarial usage and adherence, malaria infection, prevalence, risks and transmission.

**Table S3.1 – Factors influencing antimalarial drug efficacy, effectiveness, malaria infection and drug quality and supporting literature.**

| **Study** | **Influencing factor** | **Importance** |
| --- | --- | --- |
| Ataide et al., 2017 | Treatment dose | Treatment dose is a commonly used covariate in therapeutic efficacy studies [5]. |
| Danquah et al., 2009 | Malnutrition | Stunting and malnutrition in children have been identified as risk factors for antimalarial drug failure due to higher incidences of severe anaemia[6]. |
| Gething et al, 2010 | *Plasmodium falciparum* parasite rate (*Pf*PR) | *Pf*PR represents the malaria infection prevalence [7]. |
| Hay et al., 2007 | Malaria endemicity[8] | Antimalarial drug efficacy depends on the transmission intensity of a drug. |
| Hay et al. 2007  Klein et al. 2012 | Education years per capita | The level of education influence treatment-seeking behaviour and malaria treatment practices. Less educated individuals tend to use herbal remedies, buy inappropriate drugs and not adhere to dosage [9]. |
| Laufer et al., 2007  Djimde et al., 2003 | Age | Adults that have experiences several malaria infections are known to perform better in therapeutic efficacy trials than young children under 5 years [10, 11]. |
| Malaria consortium 2015 | Healthcare delivery and access; Out-of-pocket health expenditure[13] | Individuals in countries with higher out-of-pocket health expenditure are more likely to buy or receive sub-quality drugs [14]. |
| Newton et al., 2016 | Sub-standard and sub-therapeutic drugs | Sub-standard drugs are an important but largely unrecognized public health problem, which are associated with the development of resistance [9, 15]. The contribution of sub-therapeutic doses to the development of resistance was proven in the 1950s when sub-therapeutic doses of Chloroquine were added to table salt and subsequent resistance to the drug developed [15]. |
| Parobek et al., 2017 | Partner drug resistance | Partner drug resistance has proven to be a leading cause of drug failure. For example, drug resistance to mefloquine and piperaquine in Cambodia cause ACT to fail frequently [16]. |
| Stepniewska et al., 2004 | Malaria transmission intensity | Antimalarial drug efficacy depends on the transmission intensity of a drug. Areas with high malaria transmission intensity have been found to have higher drug failure rates [17]. |
| Stepniewska et al., 2004 | Admission parasite density | The odds of failure to clear parasitaemia by day 3 per 10-fold increase in admission parasite density was demonstrated to be 3.86 (95% CI 1.71 – 8.71) in high transmission areas (p = 0.001)[18]. Parasitaemia levels are a commonly adjusted variable in therapeutic efficacy studies [5]. |
| Weiss et al., 2015 | Environment/ Climate | Temperature, elevation and precipitation are the most commonly used covariates for malaria mapping[13]. |
| White, NJ, 2002 | Follow-up time | Follow-up time of therapeutic efficacy studies should be at least 28 days. Studies with shorter follow-up (i.e. < 14 days) are prone to overestimate treatment efficacy. Partner drugs with long half-lives (e.g. Mefloquine and Piperaquine) should be followed up for 42 days [19]. |
| White, NJ, 2002 | PCR-adjustment | PCR adjustment can differentiate between a reinfection and recrudescence. Studies without PCR adjustment might overestimate (i.e. reinfection is assumed for all reappearances) or underestimate (i.e. recrudescence is assumed for all reappearances) drug efficacy [19]. |
| World Health Organization 2003 | Time | Drug efficacy is known to fluctuate and change over time [20]. |
| World Health Organization 2018 | Monotherapy | Monotherapy is identified to play a crucial role in the development of drug resistance[21]. Artesunate monotherapy is thought to have contributed to the emerging drug resistance in the Greater Mekong area[22]. |

**Table S3.2 - Country level covariates; Source: Institute for Health Metrics and Evaluation (IHME)**

| **Covariate name** | **Covariate description** |
| --- | --- |
| Antenatal Care (1 visit) Coverage (proportion) | Proportion of pregnant women receiving any antenatal care from a skilled provider |
| Antenatal Care (4 visits) Coverage (proportion) | Proportion of pregnant woman receiving 4 or more antenatal care visits including 1 or more from a skilled provider |
| Education (years per capita) | Education (years per capita) |
| Health System Access (unitless) | A measure of health system access estimated using a principal component analysis of antenatal clinics, DTP3 immunization, measles immunization, hospital beds, in-facility delivery, and skilled birth attendance. |
| Health System Access 2 (unitless) | A measure of health system access estimated using a principal component analysis of antenatal clinics, DTP3 immunization, measles immunization, in-facility delivery, and skilled birth attendance. |
| DTP3 Coverage (proportion) | Fraction of children born in a given country-year who have received 3 doses of DTP3 |
| Measles Vaccine Coverage (proportion) | Percentage of population with measles vaccination |
| Skilled Birth Attendance (proportion) | Percent of women giving birth with a skilled birth attendant (mainly nurses, doctors, midwives) |
| In-Facility Delivery (proportion) | Percent of women giving birth in a health facility |
| LDI (I$ per capita) | Lag distributed income per capita (I$): gross domestic product per capita that has been smoothed over the preceding 10 years |
| Underweight (proportion <2SD weight for age, <5 years) | Proportion of children aged 0 to 59 months in a given population who fall below 2 standard deviations (SD) of the WHO 2006 standard weight-for-age curve |
| Mean BMI | Mean body mass index (kg/m^2) for males and females above age 20 |
| Elevation Under 100m (proportion) | Proportion of the population living at100m of elevation or less (2.5 arc mins) |
| Elevation 100 to 500m (proportion) | Proportion of the population living between 100m and 500m of elevation (2.5 arc mins) |
| Elevation 500 to 1500m (proportion) | Proportion of the population living between 500m and 1500m of elevation (2.5 arc mins) |
| Elevation Over 1500m (proportion) | Proportion of the population living above 1500m of elevation (2.5 arc mins) |
| Population Density (150-300 ppl/sqkm, proportion) | Proportion of the country with population density between 150 and 300 people per square kilometer |
| Population Density (300-500 ppl/sqkm, proportion) | Proportion of the country with population density between 300 and 500 people per square kilometer |
| Population Density (500-1000 ppl/sqkm, proportion) | Proportion of the country with population density between 500 and 1000 people per square kilometer |
| Population Density (over 1000 ppl/sqkm, proportion) | Proportion of the country with population density over 1000 people per square kilometer |
| Population Density (under 150 ppl/sqkm, proportion) | Proportion of the country with population density under 150 people per square kilometer |
| Population Under 30 (proportion) | Proportion of the population under age 30 |
| Population 15 to 30 (proportion) | Proportion of the population living between 0 and 15 absolute degrees latitude (2.5 arcmin grids) |
| Population Over 65 (proportion) | Proportion of the population over age 65 |
| Rainfall Population-Weighted (mm/yr) | Rainfall, population-weighted (mm/yr) |
| Rainfall (Quintiles 2-5) | Percent of the population living in the 2nd, 3rd, 4th or 5th world quintile of annual rainfall (in millimeters) ("5=most rain, 1=least rain") |
| Rainfall (Quintiles 3-5) | Percent of the population living in the 3rd, 4th or 5th world quintile of annual rainfall (in millimetres) ("5=most rain, 1=least rain") |
| Rainfall (Quintiles 4-5) | Percent of the population living in the 4th or 5th world quintile of annual rainfall, inclusive (in millimetres) ("5=most rain, 1=least rain") |
| 90th percentile climatic temperature in the given country-year. | 90th percentile climatic temperature in given country-year. |
| Malaria Lysenko PFPR 5 (Epidemic) | Proportion of the population for which malaria is epidemic, based on Lysenko |
| Malaria Lysenko PFPR 1 (Holoendemic) | Proportion of the population for which malaria is holoendemic, based on Lysenko |
| Malaria Lysenko PFPR (2 Highest Endemicity) | Proportion of the population for which malaria is holoendemic or hyperendemic, based on Lysenko |
| Malaria PFPR (rate) | *Plasmodium falciparum* parasitaemia rate |
| Year | Year |
| Age-Standardize Prevalence of Severe Anaemia | Age-Standardize Prevalence of Severe Anaemia |
| Health System Access (capped) | A measure of health system access estimated using a principal component analysis of antenatal clinics, DTP3 immunization, measles immunization, in-facility delivery, and skilled birth attendance. Maximum value for each year capped to min OECD value. |
| Interaction of malaria ITN and PFPR rate covariates | Interaction of malaria ITN and PFPR rate covariates |
| Interaction of malaria ITN and log PFPR covariates | Interaction of malaria ITN and log PFPR covariates |
| Malaria PFPR adjusted for ITN and IRS coverage | Malaria PFPR adjusted for ITN and IRS coverage |
| Hemoglobinopathies Prevalence x Excess Mortality (excluding G6PD deficiency) |  |
| Maternal education (years per capita) | Maternal education (years per capita) |
| Enhanced Vegetation Index long term average 2000-2012 (mean) | Enhanced Vegetation Index long term average 2000-2012 (mean) |
| Education age standardized | Age standardized educational attainment |
| GDP per capita base 2010 | GDP per capita base 2010 international dollars |
| Urbanicity | Urbanicity |
| Malaria incidence - from MAP estimates(AFRICA) | Year-location-age (0-5 years, 5-15 years, 15+ years) specific malaria incidence derived from MAP estimates. For continental Africa only |
| Socio-demographic Index | A measure of development estimated via principal component analysis using log-transformed LDI, TFR, and education years per capita over age 15 |
| Interaction of prevalence weighted drug resistance and malaria incidence | Interaction of prevalence weighted drug resistance and malaria incidence |
| Malaria incidence adjusted for antimalarial coverage and drug effectiveness | Malaria incidence adjusted for antimalarial coverage and drug effectiveness |
| OOP Health Expenditure per capita | Per capita out-of-pocket health expenditure |
| Fraction of OOP Health Expenditure | Fraction of out-of-pocket health expenditure out of total |
| Universal health coverage | Coverage of universal health coverage tracer interventions for prevention and treatment services, percent; created for GBD 2015 SDGs paper. |
| Healthcare access and quality index | Healthcare access and quality index |
| Malaria *Pf*PR_MAP | *P. falciparum* prevalence 1980-2016 national and subnational (added GBD2016) |
| Malaria incidence_MAP | *P. falciparum* incidence 1980-2016 national and subnational (added GBD2016) |
| Antimalarial effective treatment ratio_MAP | Proportion of effective antimalarial treatment among all treatment |
| Antimalarial effective treatment_MAP | Percent of all fevers effectively treated |
| Mean hemoglobin concentration (age-standardized) | Mean hemoglobin concentration (age-standardized) |

Website: <http://ghdx.healthdata.org/ihme_data>

**Table S3.3 – Raster layers covariates (5x5 km); Source: Malaria Atlas Project (MAP)**

| **Variable name** | **Source** | **Period covered** | **Variants** |
| --- | --- | --- | --- |
| IGBP Land cover | MODIS | 2001-2013 | Overall  Grasslands  Permanent Wetlands  Croplands  Urban and Built Up  Cropland + Natural Vegetation mosaic |
| Enhanced Vegetation Index (EVI) | MODIS derivative | 2000-2015 | Minimum of minimum  Maximum of maximum  Mean of mean  Mean of minimum  Mean of maximum  Mean of Standard Deviation |
| Surface Brightness Tasseled cap brightness (TCB) | MODIS derivative | 2000-2015 | Minimum  Maximum  Mean  Standard Deviation |
| Land Surface Temperature  Daytime  Nighttime  Diurnal Difference (i.e., daytime LST minus night-time) | MODIS derivative | 2000-2015 | Minimum of minimum  Maximum of maximum  Mean of mean  Mean of minimum  Mean of maximum  Mean of Standard Deviation |
| Accessibility to cities | MAP (19) | 2015 (Static) | ---- |
| Aridity |  | Static | Minimum  Maximum  Mean |
| Elevation | SRTM | Static | Minimum  Maximum  Mean |
| Elevation | Ferranti | Static | Maximum |
| Slope | SRTM derivative | Static | --- |
| Slope | Ferranti derivative | Static | Maximum |
| Irrigated Areas |  | Static | --- |
|  |  |  |  |
| Nighttime lights | VIIRS | Static (2012) | Global  Mean  Median |
| Nighttime lights | DMSP | Static (2010) | Global |
| Potential Evapo-Transpiration (PET) | WorldClim | Static | Global |
| Temperature Suitability Index (TSI) - for *P. falciparum* transmission | MAP (20,21) | 2000-2015 | Minimum  Maximum  Mean  Standard Deviation |
| WorldClim Temperature | WorldClim |  | Minimum  Maximum  Mean |
| Population | Worldpop | 2000-2015 | Global |
| Urban areas | Global Rural Urban Mapping Project (GRUMP) |  | Global |

Website: <https://malariaatlas.org/data-project/covariates/>

**Table S3.4 - Variables used in the final models for both artemisinin and non-artemisinin based antimalarial drug**

| **Variable** | **Artemisinin-based antimalarial drugs** | **Non- artemisinin antimalarial drugs** |
| --- | --- | --- |
| Accessibility to cities | X | X |
| Aridity | X | X |
| Landcover - Cropland + Natural Vegetation | X |  |
| Land Surface Temperature - Daytime | X | X |
| Slope (Shuttle Radar Topography Mission) | X |  |
| Elevation (Shuttle Radar Topography Mission) | X | X |
| Irrigated Areas | X | X |
| Temperature Suitability Index |  | X |
| Enhanced Vegetation Index | X | X |
| Population | X |  |
| Skilled Birth Attendance - Coverage | X |  |
| Diphtheria-tetanus-pertussis (DTP3) immunization coverage | X | X |
| Measles Vaccine Coverage |  | X |
| Coverage of Antenatal Care (1 visit) | X | X |
| Coverage of Antenatal Care (4 visit) |  | X |
| Health System Access | X | X |
| Universal health coverage | X | X |
| GDP per capita base 2010 |  | X |
| OOP Health Expenditure per capita | X | X |
| Urbanicity | X | X |
| Underweight | X | X |
| Education years per capita | X | X |
| Healthcare access and quality index | X | X |
| In-Facility Delivery | X | X |

About the covariates: We tested existence of multicollinearity and remove highly correlated covariates based on their goodness-of-fit. However, some of the covariates are modelled and there may be circularity between them, this could limit explicitly interpretation of the effect of the covariates to the response.

**Figure S3.1: Modeling Framework**

**References**

1. WHO. Guidelines for the treatment of malaria. Third edition. Geneva: World Health Organization; 2015.

2. WHO. Methods and techniques for clinical trials on antimalarial drug efficacy: genotyping to identify parasite populations. . Geneva: World Health Organization; 2008.

3. WHO. Severe falciparum malaria.: World Health Organization; 2000.

4. WHO. WHO child growth standards: length/height-for-age, weight-for-age, weight-for- length, weight-for-height and body mass index-for-age: methods and development. Geneva: World Health Organization, Multicentre Growth Reference Study Group; 2006.

5. Ataide R, Ashley EA, Powell R, Chan JA, Malloy MJ, O'Flaherty K, et al. Host immunity to Plasmodium falciparum and the assessment of emerging artemisinin resistance in a multinational cohort. Proceedings of the National Academy of Sciences of the United States of America. 2017;114(13):3515-20.

6. Danquah I, Dietz E, Zanger P, Reither K, Ziniel P, Bienzle U, et al. Reduced efficacy of intermittent preventive treatment of malaria in malnourished children. Antimicrob Agents Chemother. 2009;53(5):1753-9.

7. Gething PW, Patil AP, Smith DL, Guerra CA, Elyazar IR, Johnston GL, et al. A new world malaria map: Plasmodium falciparum endemicity in 2010. Malar J. 2011;10:378.

8. Hay SI, Guerra CA, Gething PW, Patil AP, Tatem AJ, Noor AM, et al. A world malaria map: Plasmodium falciparum endemicity in 2007. PLoS Med. 2009;6(3):e1000048.

9. Klein EY, Lewis IA, Jung C, Llinas M, Levin SA. Relationship between treatment-seeking behaviour and artemisinin drug quality in Ghana. Malar J. 2012;11:110.

10. Laufer MK, Djimde AA, Plowe CV. Monitoring and deterring drug-resistant malaria in the era of combination therapy. Am J Trop Med Hyg. 2007;77(6 Suppl):160-9.

11. Djimde AA, Doumbo OK, Traore O, Guindo AB, Kayentao K, Diourte Y, et al. Clearance of drug-resistant parasites as a model for protective immunity in Plasmodium falciparum malaria. Am J Trop Med Hyg. 2003;69(5):558-63.

12. Laufer MK. Monitoring antimalarial drug efficacy: current challenges. Curr Infect Dis Rep. 2009;11(1):59-65.

13. Weiss DJ, Mappin B, Dalrymple U, Bhatt S, Cameron E, Hay SI, et al. Re-examining environmental correlates of Plasmodium falciparum malaria endemicity: a data-intensive variable selection approach. Malar J. 2015;14:68.

14. consortium M. Universal health coverage and malaria, neglected tropical diseases and child health. 2015.

15. Paul N. Newton ClCPJG. A link between poor quality antimalarials and malaria drug resistance? Expert Review of Anti-infective Therapy. 2016;14:6:531-3.

16. Parobek CM, Parr JB, Brazeau NF, Lon C, Chaorattanakawee S, Gosi P, et al. Partner-Drug Resistance and Population Substructuring of Artemisinin-Resistant Plasmodium falciparum in Cambodia. Genome biology and evolution. 2017;9(6):1673-86.

17. Stepniewska K, Taylor WR, Mayxay M, Price R, Smithuis F, Guthmann JP, et al. In vivo assessment of drug efficacy against Plasmodium falciparum malaria: duration of follow-up. Antimicrob Agents Chemother. 2004;48(11):4271-80.

18. Stepniewska K, Ashley E, Lee SJ, Anstey N, Barnes KI, Binh TQ, et al. In vivo parasitological measures of artemisinin susceptibility. J Infect Dis. 2010;201(4):570-9.

19. White NJ. The assessment of antimalarial drug efficacy. Trends in Parasitology. 2002;18(10).

20. WHO. Assessment and monitoring of antimalarial drug efficacy for the treatment of uncomplicated falciparum malaria. Geneva: World Health Organization; 2003.

21. Dondorp AM, Nosten F, Yi P, Das D, Phyo AP, Tarning J, et al. Artemisinin resistance in Plasmodium falciparum malaria. N Engl J Med. 2009;361(5):455-67.

22. WHO. Responding to antimalarial drug resistance Geneva: World Health Organization; 2018 [Available from: <http://www.who.int/>.

**Section 4 – Median and interquartile range of antimalarial effectiveness**

The median effective levels and the interquartile range (IQR) for the arteminsinin-based antimalarial drugs for the periods 1991-2000, 2001-2005, 2006-2010, 2011-2015, and 2016-2019 are presented in Figures S4.1 and S4.2. These were obtained from 100 posterior samples drawn from the predictive distribution. IQR maps for the two drugs are presented in Figures S4.3 and S4.4


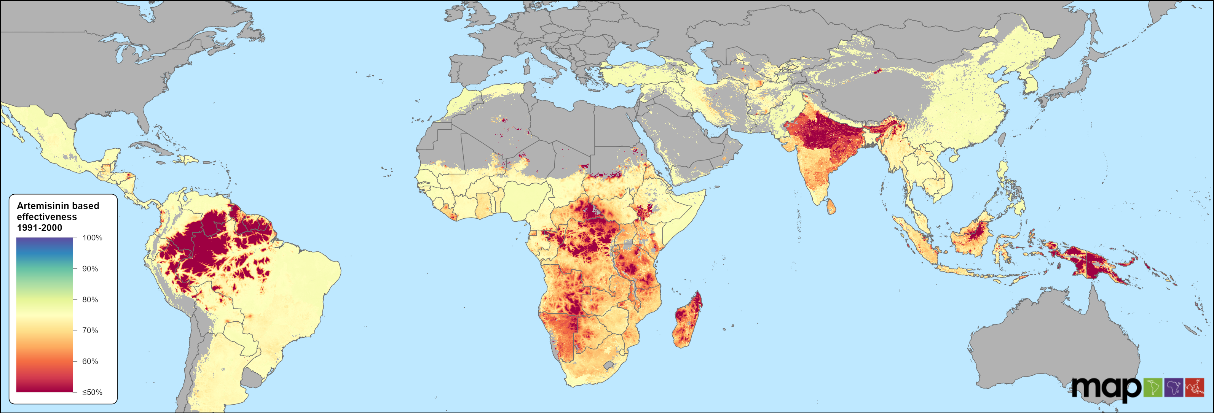

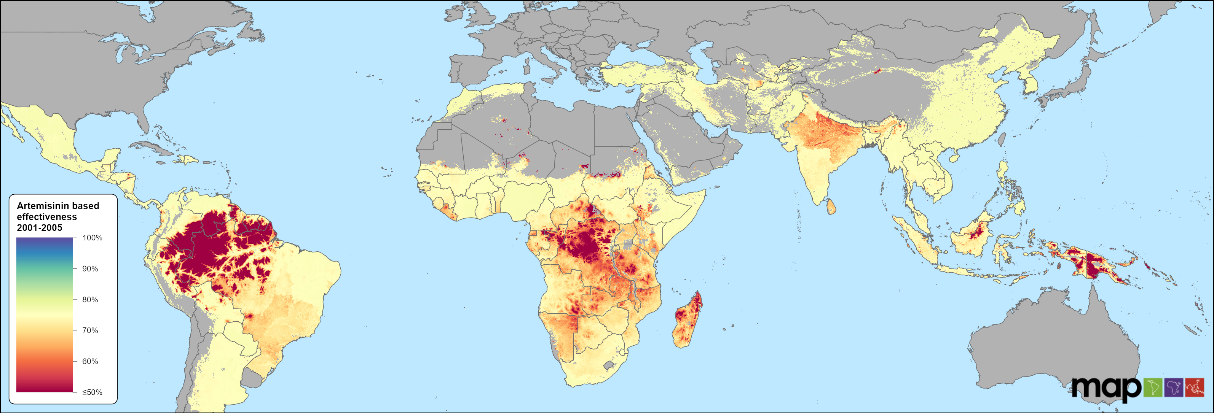

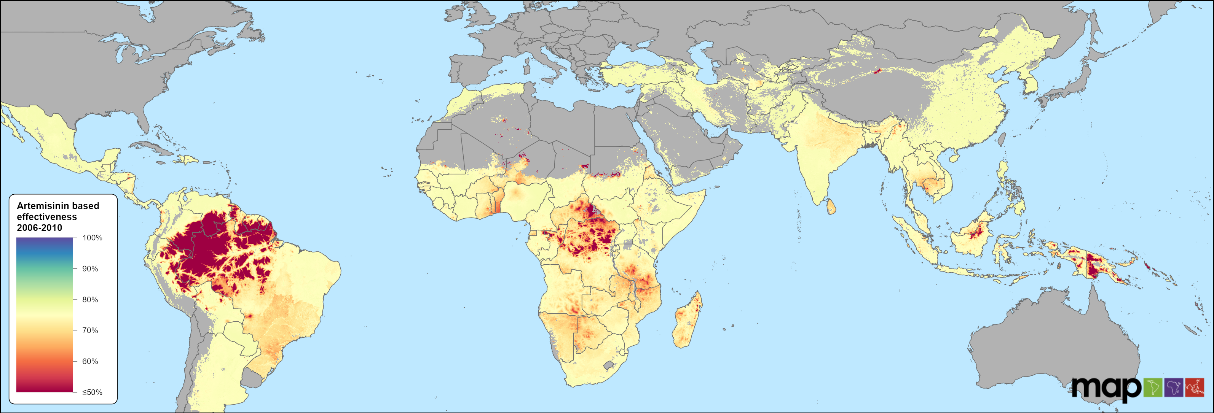

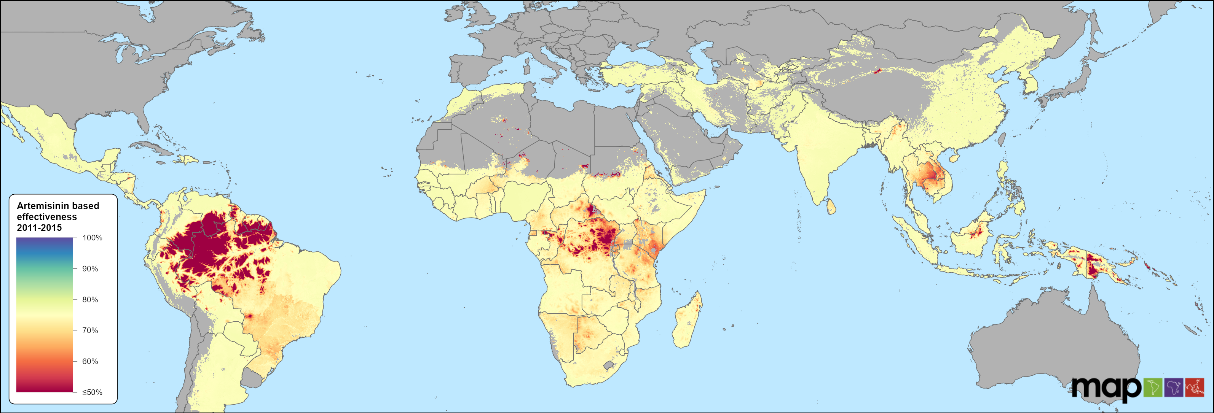

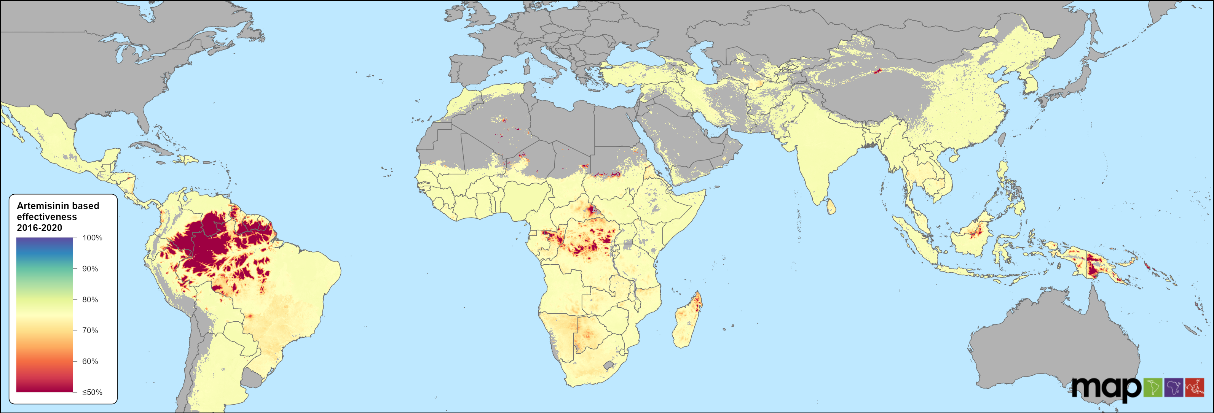


**Figure S4.1: Spatiotemporal distribution of median effectiveness of artemisinin-based antimalarial drugs for periods 1991-2000, 2001-2005, 2006-2010, 2011-2015, and 2016-2019.**


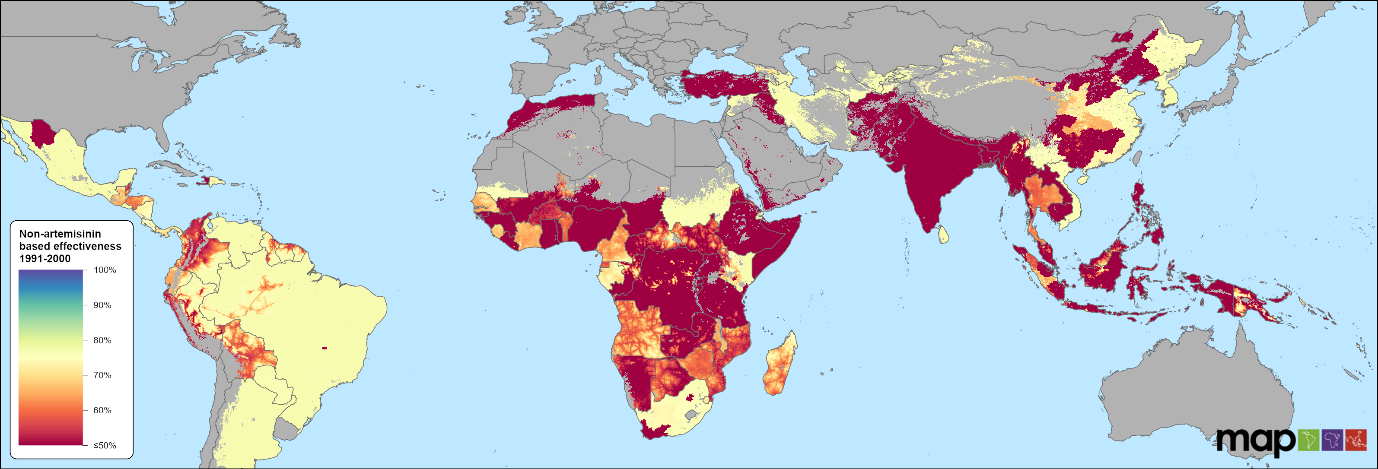

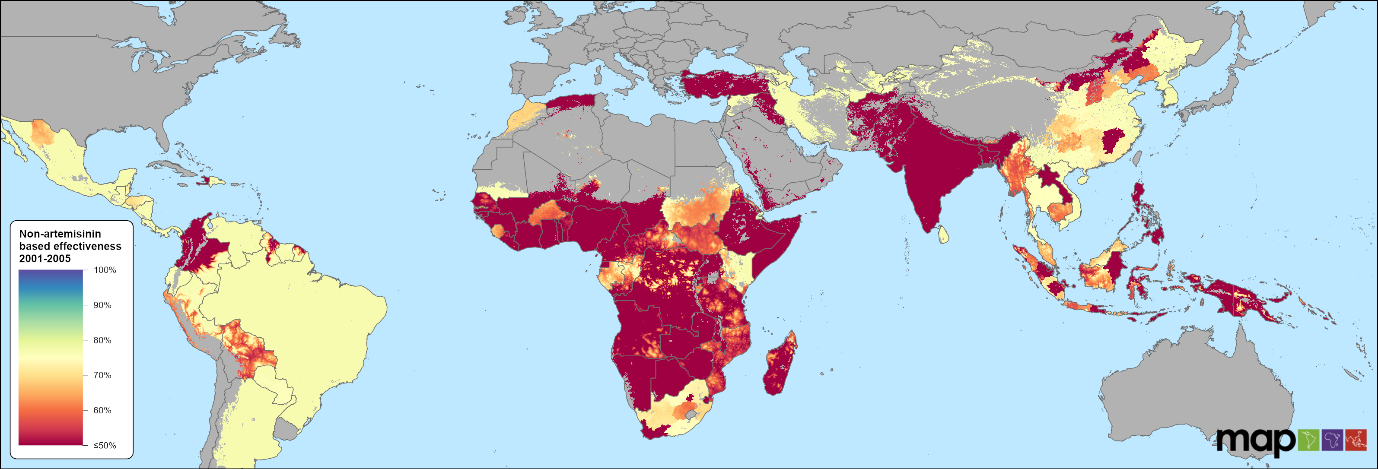

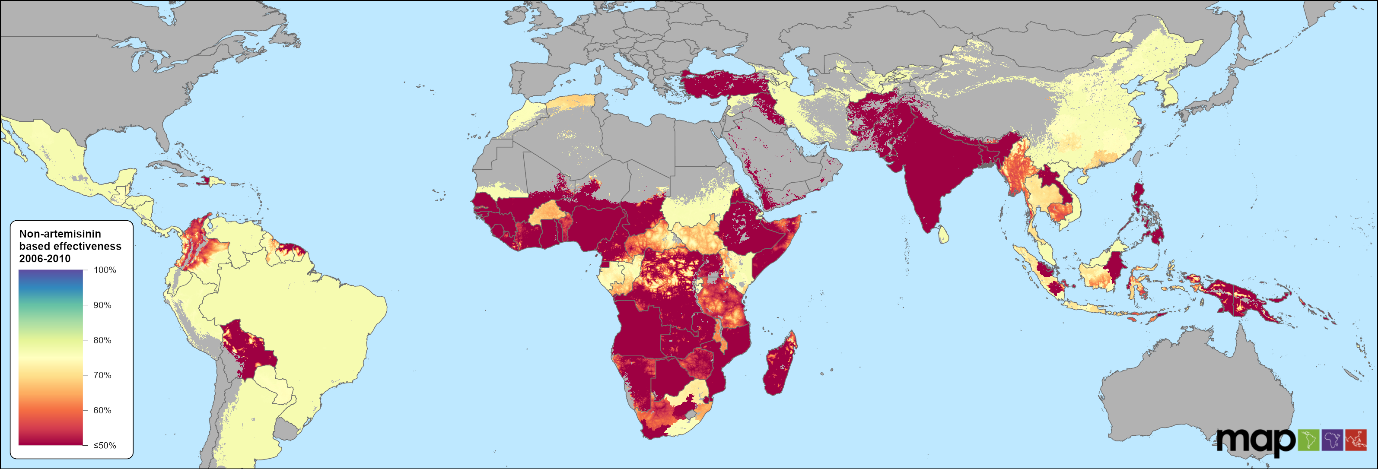

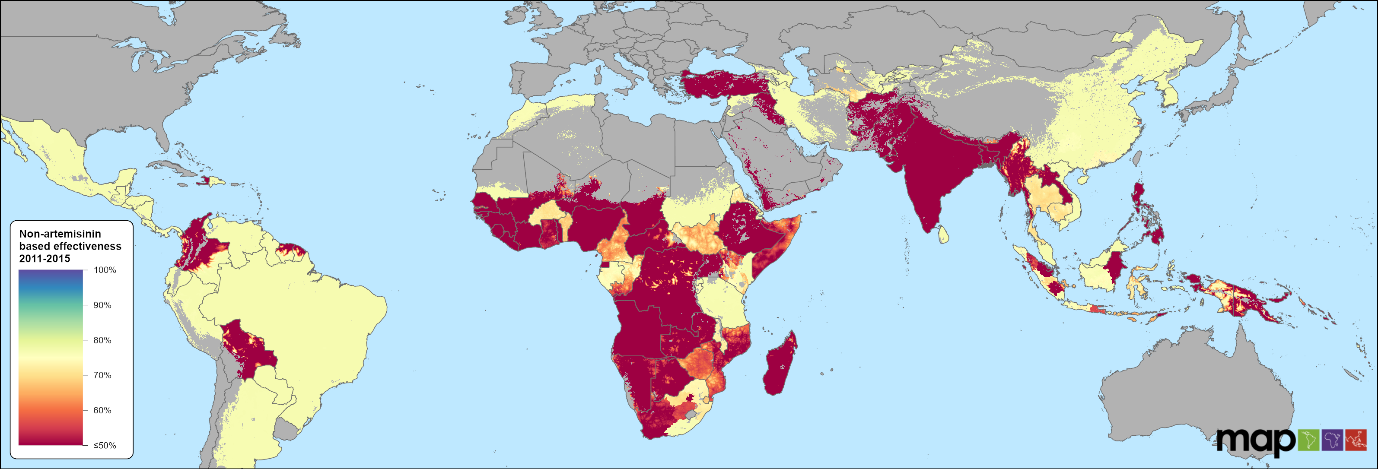


**Figure S4.2: Spatiotemporal distribution of median effectiveness of non-artemisinin antimalarial drugs for periods 1991-2000, 2001-2005, 2006-2010, and 2011-2015.**

**
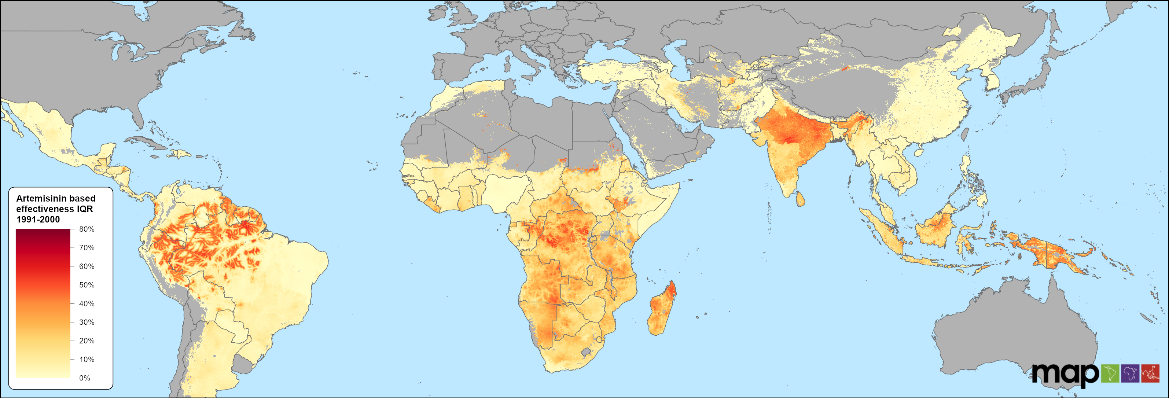

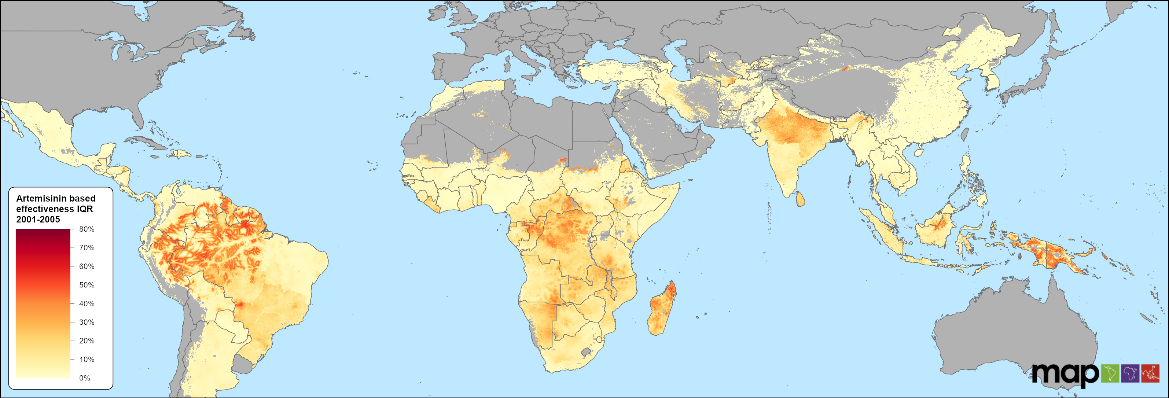

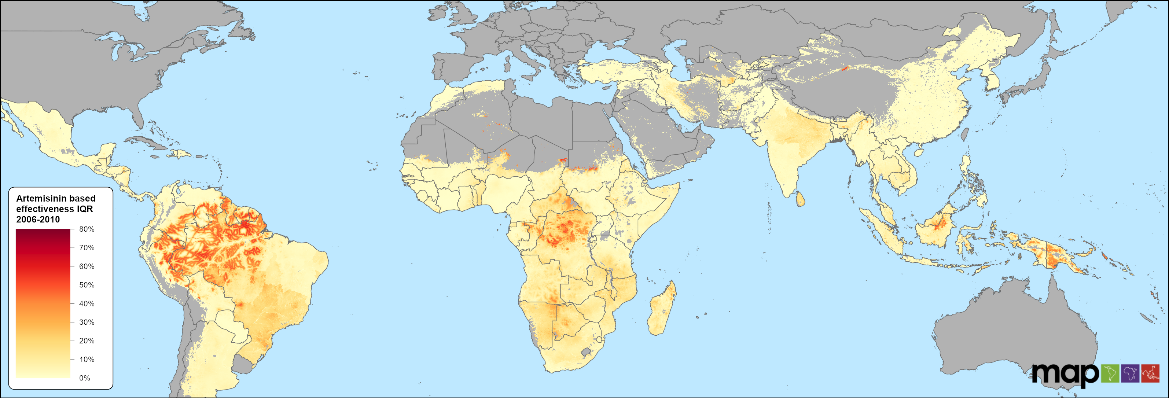

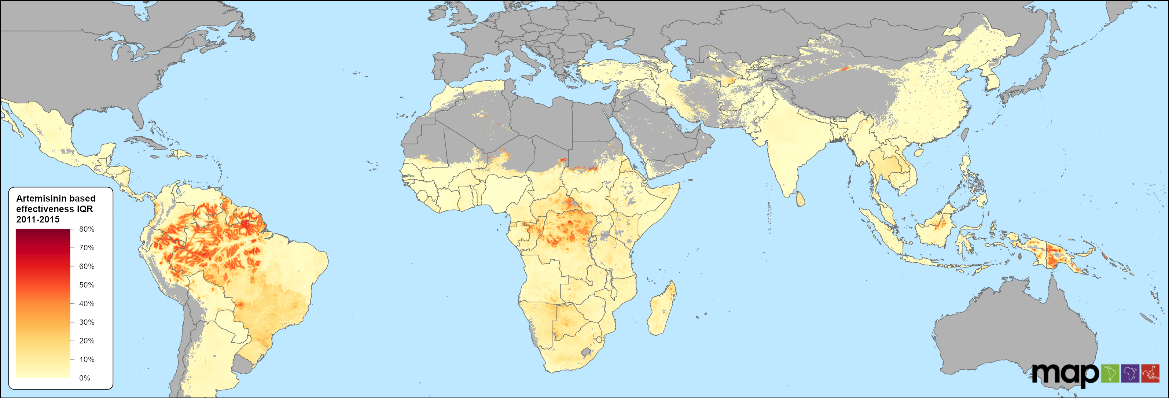

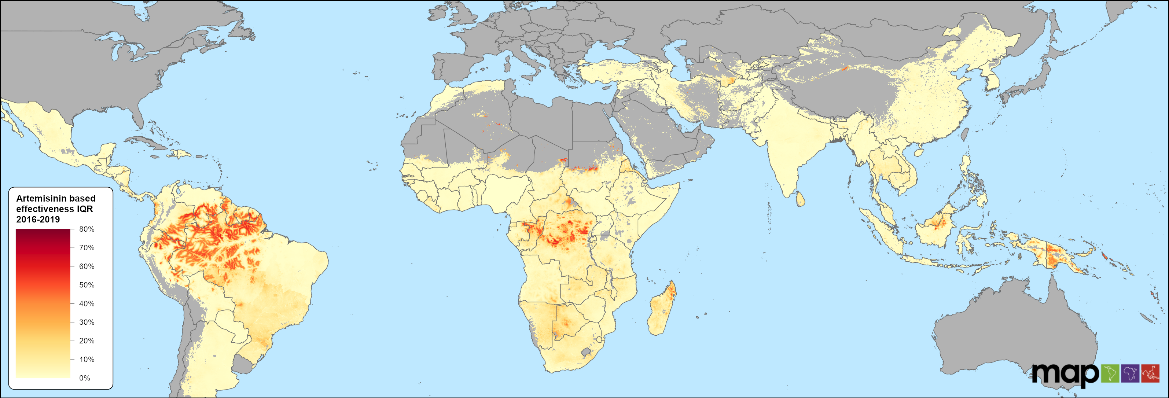
**

**Figure S4.3: Maps of interquartile range (IQR) for artemisinin-based antimalarial drug effectiveness for the 1991-2000, 2001-2005, 2006-2010, 2011-2015, and 2016-2019.**

**
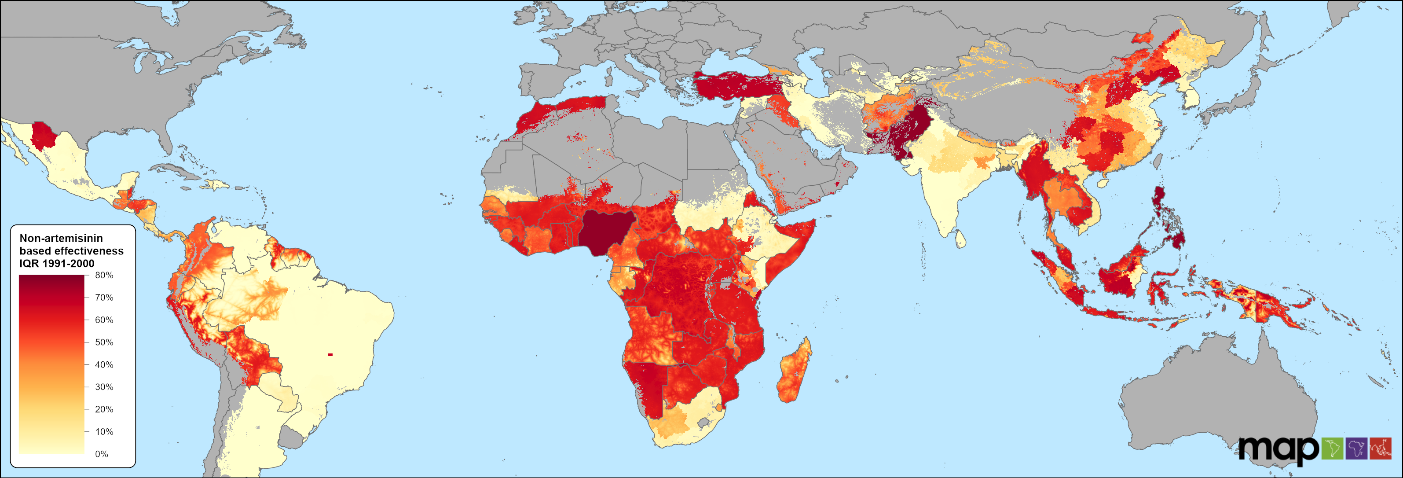

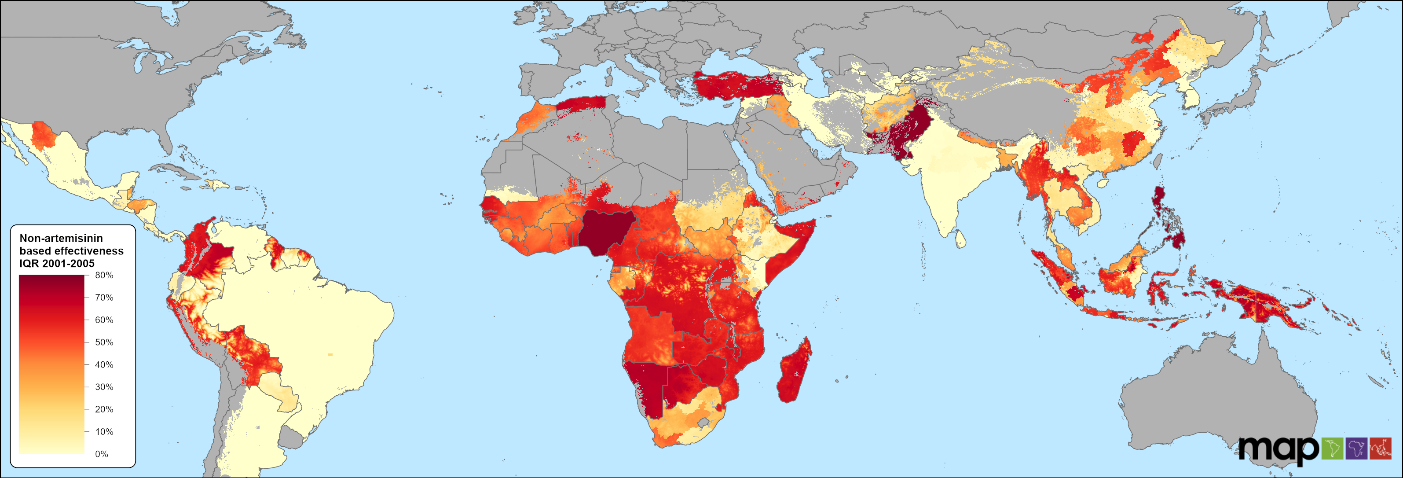

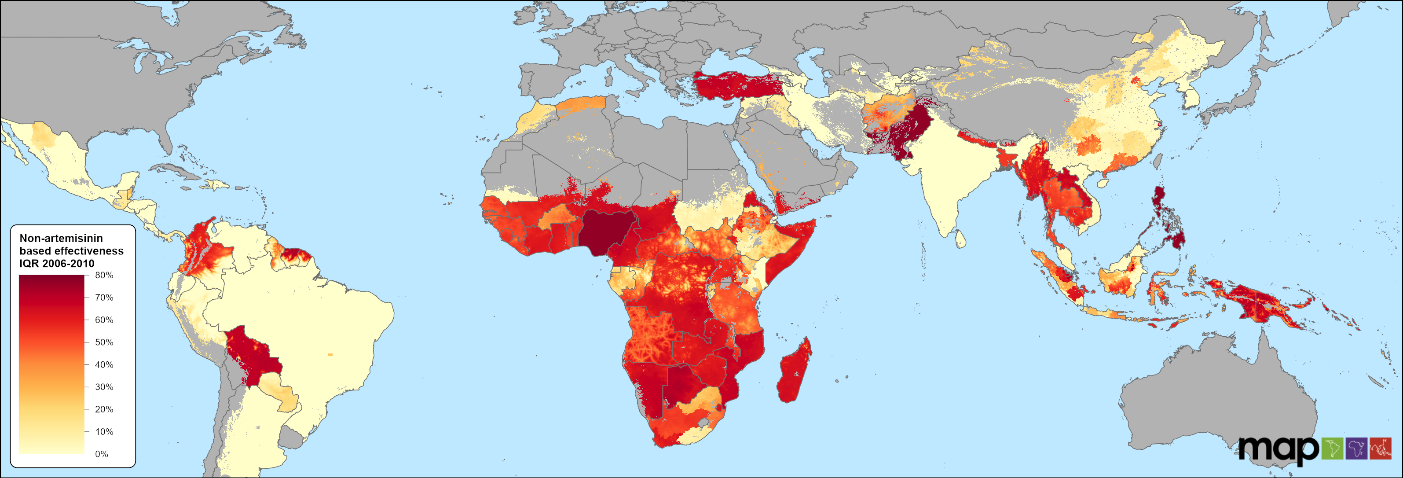

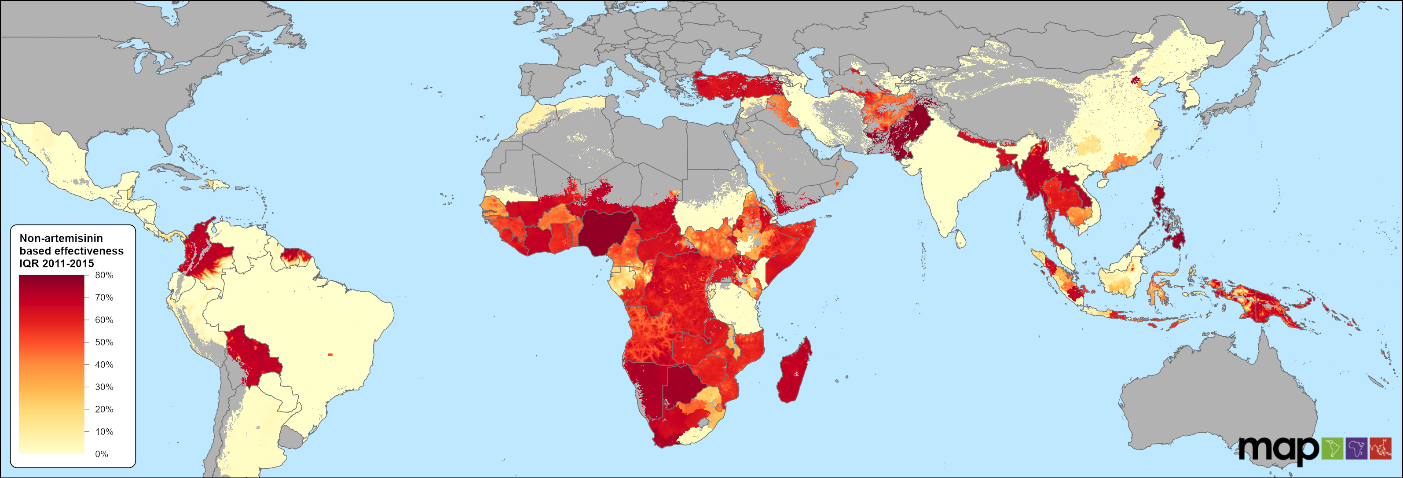
Figure S4.4.: Maps of interquartile range (IQR) for non-artemisinin-based antimalarial drug effectiveness for the periods 1991-2000, 2001-2005, 2006-2010, and 2011-2015.**

1. * According to WHO criteria^1^ [↑](#footnote-ref-1)
2. [↑](#footnote-ref-2)
